# Estimating three-dimensional outflow and pressure gradients within the human eye

**DOI:** 10.1371/journal.pone.0214961

**Published:** 2019-04-09

**Authors:** David W. Smith, Chang-Joon Lee, William Morgan, Bruce S. Gardiner

**Affiliations:** 1 Faculty of Engineering and Mathematical Sciences, The University of Western Australia, Perth, Australia; 2 College of Science, Health, Engineering and Education, Murdoch University, Murdoch, Western Australia, Australia; 3 Lions Eye Institute, The University of Western Australia, Perth, Australia; University of Nottingham, UNITED KINGDOM

## Abstract

In this paper we set the previously reported pressure-dependent, ordinary differential equation outflow model by Smith and Gardiner for the human eye, into a new three-dimensional (3D) porous media outflow model of the eye, and calibrate model parameters using data reported in the literature. Assuming normal outflow through anterior pathways, we test the ability of 3D flow model to predict the pressure elevation with a silicone oil tamponade. Then assuming outflow across the retinal pigment epithelium is normal, we test the ability of the 3D model to predict the pressure elevation in Schwartz-Matsuo syndrome. For the first time we find the flow model can successfully model both conditions, which helps to build confidence in the validity and accuracy of the 3D pressure-dependent outflow model proposed here. We employ this flow model to estimate the translaminar pressure gradient within the optic nerve head of a normal eye in both the upright and supine postures, and during the day and at night. Based on a ratio of estimated and measured pressure gradients, we define a factor of safety against acute interruption of axonal transport at the laminar cribrosa. Using a completely independent method, based on the behaviour of dynein molecular motors, we compute the factor of safety against stalling the dynein molecule motors, and so compromising retrograde axonal transport. We show these two independent methods for estimating factors of safety agree reasonably well and appear to be consistent. Taken together, the new 3D pressure-dependent outflow model proves itself to capable of providing a useful modeling platform for analyzing eye behaviour in a variety of physiological and clinically useful contexts, including IOP elevation in Schwartz-Matsuo syndrome and with silicone oil tamponade, and potentially for risk assessment for optic glaucomatous neuropathy.

## Introduction

Glaucoma is the most significant cause of irreversible blindness world-wide, with some 70 million people affected [1]. While glaucoma is a group of diseases, there is a crucially important association between the initiation and progression of glaucoma and an elevated intraocular pressure (IOP). The only proven treatment of glaucoma is the reduction of IOP [1–3].

There are many different theories as to the cause of glaucoma, including vascular dysfunction [4, 5], metabolic and mitochondrial dysfunction [6], and various kinds of mechanical dysfunction [7, 8]. Nevertheless, the magnitude of IOP elevation is correlated with the rate of ganglion cell loss [1, 9]. For this reason alone, elevated IOP is believed to play a fundamental role in glaucoma progression [2, 8], though it is usually suspected that a wide variety of additional factors play contributing roles [1, 8, 10].

Some authors go further and argue that elevated IOP is not only correlated with glaucoma, but in fact causes glaucoma. For example, Jonas et al. observes that numerous studies have shown that high IOP leads to glaucomatous optic neuropathy, that ‘*lowering IOP stops the progression*’ of the neuropathy [11], and that increasing IOP ‘*again causes progression*’ of the neuropathy [11]. This leads Jonas et al. to observe that these observations fulfil ‘Koch’s postulates’, which are employed to prove a microbe is causative of a disease, and so argues that based on this accepted basis for establishing causation in the biological sciences, the existing data supports the statement that elevated IOP is causative of glaucoma.

Using criteria for ‘causal inference’ in epidemiology, a similar conclusion is reached by Bahrami [12]. To satisfy causation based on epidemiological evidence, there needs to be a plausible biological ‘mechanism’ linking elevated IOP with glaucoma. Such an experimentally observable ‘link mechanism’ does exist, for it is extensively documented that elevated IOP causes interruption of axonal transport at the optic nerve head in the axons of retinal ganglion cells [6, 13–18]. Retinal ganglion cell death follows disruption of retinal ganglion cell axonal transport [6, 19], which explains vision loss with glaucoma.

Searching for a possible cause of axonal transport disruption in retinal ganglion cells, one observation is that elevated IOP causes displacement and distortion of the laminar cribrosa in the optic nerve head [20]. Indeed, readily observable ‘cupping’ of the superficial layer in the optic nerve is one of the hallmarks of glaucoma [21, 22]. The distortion of laminar cribrosa is then believed to cause imprecisely defined ‘damage’ to the retinal ganglion cell axons passing through it [8]. This imprecisely defined axonal damage leads to disruption of axonal transport, again by ill-defined mechanisms, together with a number of other possible adverse effects on astrocytes, endothelial cells and connective tissue cells located in the optic nerve head and particularly in the laminar cribrosa [8]). However the mechanism or mechanisms responsible for axonal transport disruption remain uncertain [6].

A potentially troubling issue with the proposal that elevated IOP causes glaucoma is the evidence that a significant fraction of the glaucomatous population have normal IOP (often defined as an IOP less than 21 mm Hg). While elevated IOP does cause glaucoma [1, 9, 11], it is now recognised that so-named ‘normal tension glaucoma’ (NTG) may have an incidence as high as 30% to 40% in Caucasian populations, and even higher incidences in specific racial and ethnic groups [23, 24]. While some suggest this evidence points to causes of glaucoma other than elevated IOP, other researchers favour a simple modification to the ‘elevated IOP causes glaucoma’ hypothesis.

In a series of papers, Morgan et al. describes a modified hypothesis; that the ‘translaminar pressure gradient’ (TLPG) causes glaucoma. Using a four compartment model, Morgan et al. argue that displacement and strain of the laminar cribrosa is due to the fluid pressure difference across the laminar cribrosa [21, 25]. In other words, it is proposed that it is the TLPG within the optic nerve head [7, 11, 23, 26] that causes displacement and distortion of the laminar cribrosa, which then leads to axonal damage. Roy Chowdhury and Fautsch explain: ‘*the axons move from a higher IOP to a comparatively low retrobulbar pressure’* [23].

But in addition to the TLPG causing displacement and distortion of the laminar cribrosa, the TLPG also causes an interstitial fluid flow across the optic nerve head and through the optic nerve tissues. Interstitial fluid flow through the optic nerve head is evidenced by the movement of molecules from the vitreous humor into the interstitial fluid of the optic nerve [27, 28]. In addition to interstitial fluid flow, Band et al. proposed there is fluid flow through axoplasm in the retinal ganglion cells [29]. Once again, it is postulated that the link between TLPG and glaucoma is the disruption of axonal transport within the optic nerve head, but this time glaucoma is caused by the fluid flow along axons induced by the TLPG, which leads to retinal ganglion cell degeneration and loss of vision [6, 16, 26, 29]. We note that Band et al. proposes a specific mechanism to explain disruption of axonal transport, namely, that flow along the axon causes ATP ‘washout’, which reduces the supply of ATP for the molecular motors that enable axonal transport [29].

**Fig 1:**
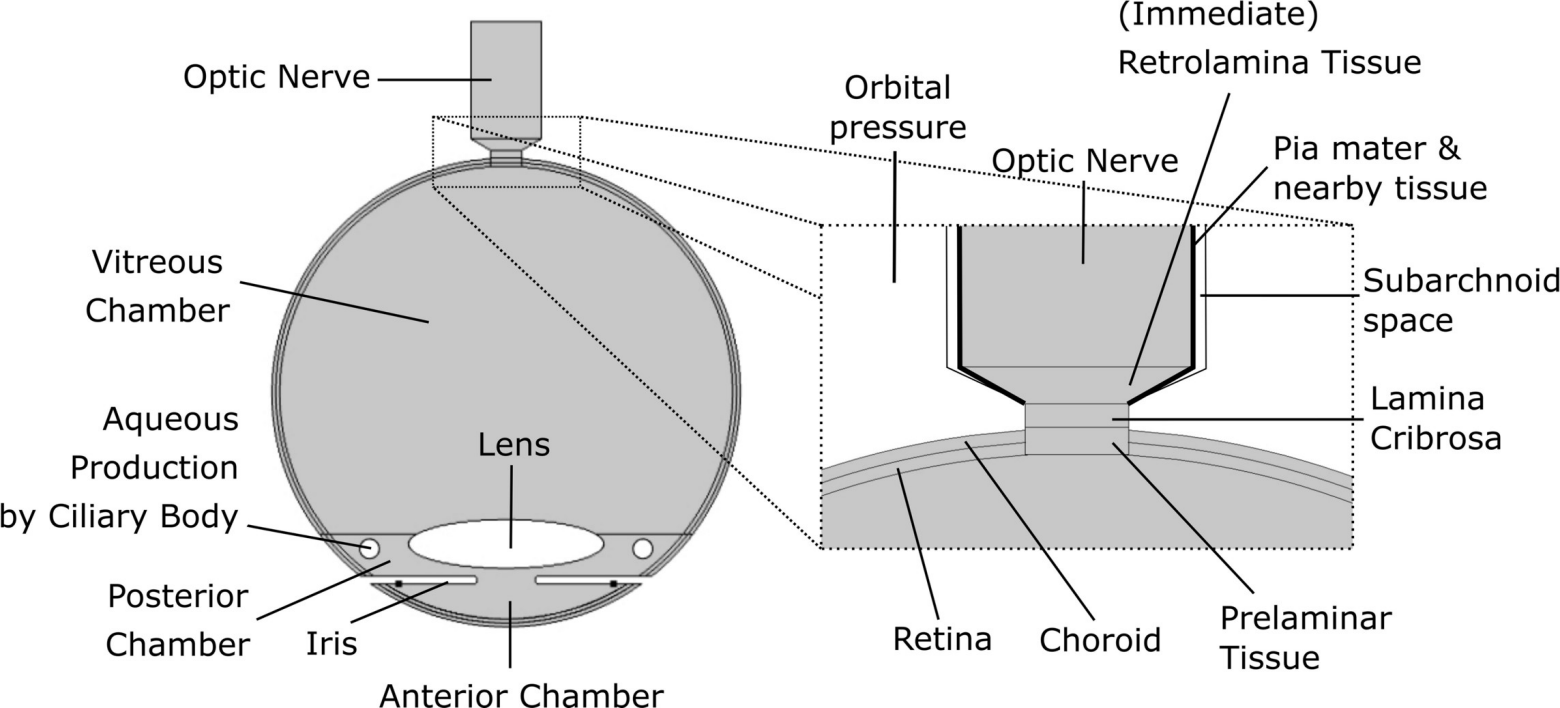
Schematic depiction of geometry and tissues of the human eye.

The theory that the TLPG causes glaucoma provides the primary motivation for developing the three dimensional (3D) pressure-dependent outflow model presented here. So after we build and calibrate the new 3D eye outflow model, we attempt to quantify the TLPG in the human eye under a variety of states. By definition, the average translaminar (cribrosa) pressure gradient depends on the thickness of the laminar cribrosa and the fluid pressures upstream and downstream of the laminar cribrosa (which in turn are related to the IOP and the average immediate retrolaminar pressure—see Fig 1). It has been found experimentally that above a ‘floor pressure’ of about 2.6 to 4.4 mm Hg, which is set by the ‘orbital pressure’ the tissues behind the eye [25, 26], the average retrolaminar pressure is largely determined by the pressure in the cerebrospinal fluid (CSF) pressure in the sub-arachnoid space surrounding the optic nerve [11, 23, 25, 26, 30]. Providing subarachnoid CSF pressures around the optic nerve are above the orbital pressure, lowering subarachnoid CSF pressure around the optic nerve will lower retrolaminar pressure. And if we assume an invariant normal IOP, it is now apparent that lowering the retrolaminar pressure will increase the average TLPG, and so elevate the risk of glaucoma.

While this modified hypothesis (‘elevated TLPG causes glaucoma’) may explain some normal tension glaucoma, this still remains to be confirmed, and clearly some believe that it may not explain all NTG [24, 31, 32]. Nevertheless, it is apparent that this modified hypothesis is very appealing, as it brings a conceptual organization to a very large amount of biologic and clinical data collected over the last 50 years. The modified hypothesis not only embraces all the previous observations that elevated IOP causes glaucoma, and potentially explains NTG due to a combination of a small elevation of IOP and lowering of retrolaminar pressure, it also explains why raising retrolaminar pressure, as occurs in idiopathic intracranial hypertension and during long-term space flight, reduces the TLPG, which given sufficient time leads to papilledema [7, 23, 29, 31, 33].

The fact that all these observations can be consistently and plausibly explained through a consideration of the TLPG, suggests a reasonably high level of confidence in the hypothesis that ‘elevated TLPG causes glaucoma’. Clearly the magnitude of the TLPG is of crucial importance in the initiation and progression of glaucoma, and so in this paper we wish to quantitatively explore our model predicted TLPG for the human eye at different times of day and in different body positions.

If accurate predictions are to be generated, the accuracy of the computational model is crucially important, so we first explain the development of the computational flow model in detail. In a previous paper we developed (and tested against a large amount of experimental data), a new ‘ODE’ (ordinary differential equation) model for IOP prediction that employs a new pressure-dependent outflow facility [34]. Assuming a constant rate of aqueous production, this ODE model predicts that if the pressure dependent outflow facility decreases too quickly as IOP rises, then IOP will become elevated. Further, the IOP may become unstable, meaning the eye will experience large pressure fluctuations for small variations in aqueous production rate. The pressure dependent outflow model predicts that aqueous production at the ciliary body in a normal, aged eye is around 6.0 microlitres/min [34], approximately twice the rate of aqueous flow usually reported in the literature [35, 36]. A detailed consideration of all outflow pathways from the eye reveals approximately half of the aqueous production may exit through the anterior chamber of the eye, travelling through the so-named ‘unconventional’ (uveoscleral) flow pathway and the ‘conventional’ (trabecular meshwork) flow pathway (where it may be measured clinically, and is consistent with reported aqueous production rates) [34]. Our model also has about half of the aqueous production traveling posteriorly through the vitreous humor, exiting across the retinal pigment epithelium, but this outflow has only been measured mainly in a research context, and only occasionally in the clinic in special circumstances [34].

In this paper we set this new ODE-pressure-dependent outflow model for the human eye in an (axisymmetric) 3D ‘partial differential equation’ (PDE), porous media model of the human eye. The ‘porous media’ aspect of the model allows us to predict interstitial flow induced by a pressure gradients across tissues in the eye. We carefully calibrate this model, including the systematic estimation of the hydraulic conductivity of tissues (a measure of resistance to interstitial fluid flow for a given pressure gradient) making up the optic nerve head. This calibrated 3D flow model enables us to visualize the advective flow patterns throughout the chambers of the eye, and to quantify the pressure gradients across the vitreous between the anterior chamber and the pressure close to the optic disc (which is the relevant IOP for quantifying the TLPG). For constant aqueous production, we first quantify how the IOP changes as ‘flow resistances’ are sequentially introduced (e.g. the retina, the vitreous and the optic nerve head). Alternatively assuming constant anterior chamber IOP, we quantify how much the additional flow resistances alter aqueous production. We also quantify the expected IOP if we change the fraction of outflow leaving the eye through the anterior pathways.

Having established the 3D flow model we perform a limited parameter sensitivity analysis. We then seek to demonstrate the conceptual validity of our new 3D flow model that has about half the outflow passing anteriorly and half the outflow passing across the retinal pigment epithelium. To do this, we assume normal anterior outflow, and then show how the new 3D flow model can explain elevated IOP in the presence of a silicone oil tamponade in the vitreous chamber. To do this, we simply assume contact between the silicone oil and the retina blocks outflow across the retinal pigment epithelium beneath the silicone oil. We then do the converse, and assume normal outflow across the retinal pigment epithelium, and show how the new 3D flow model can explain elevated IOP in Schwartz-Matsuo syndrome. To do this, we simply assume a reduction in outflow facility commensurate with the clinically measured reduction in outflow facility. Our success in modeling these two quite different but theoretically complementary conditions helps build confidence in validity of the 3D flow model.

We employ the 3D flow model to quantify daytime and nocturnal TLPG in both upright and supine postures. Using ratios of TLPGs, we estimate factors of safety against acute interruption of axonal transport. We can calculate the fraction of aqueous production flowing through the optic nerve head, and most importantly, its average fluid velocity. This flow velocity enables us to estimate ‘drag force’ on the cargos being carried by molecule motors along microtubules. We examine the proposition that an increased TLPG causes an increase in the fluid flow velocity through the axoplasm, and this increased fluid flow velocity results in an increased drag force on neurofilaments being transported in the retrograde direction by dynein molecular motors. By equating the dynein motor stall force to fluid flow velocity, we can again define an independent factor of safety against acute interruption of axonal transport. Pleasingly, both the TLPG analysis and the flow analysis reveal almost identical factors of safety for acute interruption of axonal transport.

**Fig 2:**
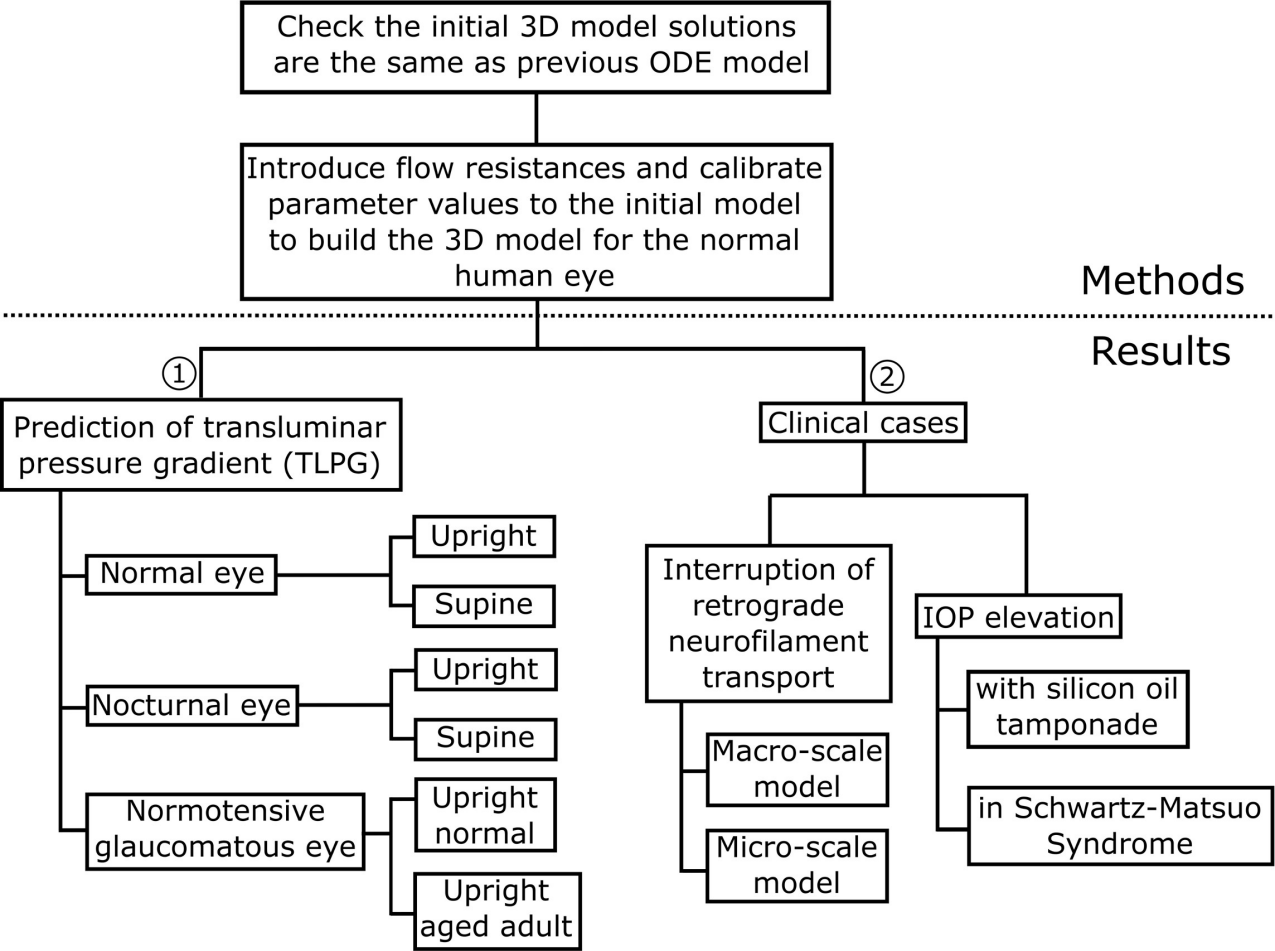
Overview of model development (see Methods section), investigations and case-studies undertaken in this paper (see Results section).

We begin by describing the method for eye outflow analysis, move on to parameter selection, and finally to calculating the behaviour of the eye in variety of different physiological states, both normal and abnormal. For reference, an overview of our program of investigations and case studies is summarised in Fig 2.

## Method

### Governing equations and boundary conditions

In this section we describe the governing equations and boundary conditions employed for the model. We solve the steady-state fluid mass balance equation within a rigid porous media, viz,

$$\nabla \cdot v_{d}=s_{i}$$ (1)

where $v_{d}$ is the Darcy velocity (m/s) and $s_{i}$ are fluid sources or sink (m^3^/s-m^3^). We employ Darcy’s law as the constitutive equation for flow through saturated tissues. The Darcy velocity is that fluid velocity when multiplied by the gross cross-sectional area normal to the flow gives the actual fluid discharge rate. The Darcy velocity is calculated using,

$$v_{d}=\frac{k_{\mathrm{int}}}{\mu_{f}}\nabla p=k\nabla p$$ (2)

where p is the spatially variable fluid pressure within eye tissues (Pa), $k_{\mathrm{int}}$ is the intrinsic hydraulic conductivity (m^2^), $\mu_{f}$ is the fluid viscosity (Pa-s) and k is the hydraulic conductivity (m^2^/Pa-s). We mention that the ‘hydraulic conductivity’ is exactly the same as fluid ‘permeability’ (which should not to be confused with diffusion permeability). Hydraulic conductivity is adopted in American usage and permeability adopted in English usage.

The Darcy velocity is related to the average true fluid velocity ($v_{t}$) by,

$$v_{t}=v_{d}/n_{f}$$ (3)

where $n_{f}$ is the porosity of the fluid phase. It is assumed that averaging volumes and areas are sufficiently large so that volumetric porosity is always the same as areal porosity.

All the model parameters described above are spatial variable, and may take on different values for different tissues. In principle, the hydraulic conductivity is a second order tensor and so it may also vary with direction. We have assumed isotropic material properties, and so the second order hydraulic conductivity tensor reduces to a scalar quantity.

The assumption of isotropic material properties in the model is certainly reasonable for vitreous humor and retina, but the choice is less clear for tissues such as the optic nerve, which has obvious textural anisotropy. Nevertheless, the assumption of isotropy in the optic nerve appears to be reasonable in the context of experimental data showing the fluid pressure in the optic nerve is essentially constant in the radial direction through the nerve, with a rapid pressure change at the periphery of the nerve [37]. This data means that any reduced hydraulic conductivity in the radial direction relative to the hydraulic conductivity in the longitudinal direction, is actually dominated by the resistance to outflow across the pial membrane and adjacent tissues, close to the nerve surface. For this situation, the assumption of an isotropic hydraulic conductivity for the optic nerve is reasonable.

To solve this system of equations at steady state we require boundary conditions on the domains. In addition to a specified aqueous production rate and pressure on some surfaces, the pressure dependent flux boundary conditions we adopt here are those derived in [34]. In the context of the porous media model, the pressure dependent outflow boundary conditions are given by,

$$v_{dn}=\frac{C_{i}^{SL}}{A_{i}\alpha }(e^{-\alpha p_{T}}-e^{-\alpha (p-p_{back})})$$ (4)

where $v_{dn}$ is the Darcy velocity normal to the outflow surface, $C_{i}^{SL}$ is the surface hydraulic conductivity for the *i*^th^ surface (μL/min/mmHg), $A_{i}$ is the area (m^2^) of the *i*^th^ surface, *α* is an exponential decay constant (mmHg^-1^) (which we chose as constant for the whole eye), $p_{T}$ is the no-flow intraocular pressure (mmHg), $p_{back}$ is the ‘all-round’ backpressure (mmHg) (which by definition is constant for the whole eye), and p (mmHg) is the IOP on the outflow surface (which varies across the eye). Eq (4) may be viewed as a special type of Robin boundary condition, where the difference between the boundary pressure and the ‘reference’ pressure is predetermined i.e. it is modelled as an exponential decreasing relationship as shown in Eq (4), which depends on the parameters $C_{i}^{SL}$, *α*, $p_{T}$ and $p_{back}$.

In general, all these quantities may vary with tissue or anatomical location within the eye, and in general they are also spatially variable over each surface. However, while we allow the quantities to vary with tissue or anatomical location, in the absence of any data to suppose otherwise, we assume that the variables are constant over each surface.

We solve the porous media flow equations and boundary conditions for a geometry that approximates an average human eye, using a finite-element solver called Comsol Multiphysics (version 5.0, COMSOL, Burlington, MA). All simulations were carried out using a Dell PC with an Intel Core i7 3.40-GHz CPU running Windows 7 Professional. The solution time for the 3D flow models using 6,000 finite elements with cubic interpolation functions typically averaged around three seconds.

### Geometry for 3D eye flow model with pressure dependent outflow

The human eye is represented as a spherical structure with an internal diameter of 23.6 mm (S1 Table). Fluid flows from the ciliary body (represented for simplicity by a torus in the region of the ciliary body) into the posterior chamber of the eye, and then to outflow pathways anteriorly and in a posterior direction across the retinal pigment epithelium. The lens and iris are treated as impermeable for simplicity. We note that these simplifying assumptions (e.g. representing the ciliary body as a torus) do not materially influence the results of the analysis presented here.

The optic nerve has been shifted about 2.7 mm from its normal ‘off-centre’ location to the ‘axis of symmetry’ for computational convenience. That is, the optic nerve is shifted to the deepest point of the eyeball in our model (see Table 3 in [38] for details of the distance from optic nerve to deepest point in the eyeball for normal eyes). This small adjustment does not materially influence the normal eye outflow model, and greatly simplifies the computational model, as axisymmetry of the problem domain can then be exploited.

The optic nerve is about 50 mm long in humans, but only about 6 mm of the optic nerve is included in the model, as this is deemed sufficient to understand the interaction of the optic nerve with fluid flow through the optic nerve head into the subarachnoid space around the optic nerve. We note that the ‘optic nerve head’ is made up of surface layer of retinal ganglion cell axons, the prelaminar tissue and the laminar cribrosa, before axons enter the immediate retrolaminar tissue, which is the proximal part of the optic nerve where the percentage of myelinated axons is increasing immediately behind the laminar cribrosa (see Fig 1) [39]. After passing through the laminar cribrosa, the axons in the optic nerve become myelinated and so the optic nerve’s diameter expands considerably post laminar cribrosa from a diameter of about 1.6 mm at the optic disc to a diameter of 3.2 mm for the optic nerve.

**Fig 3:**
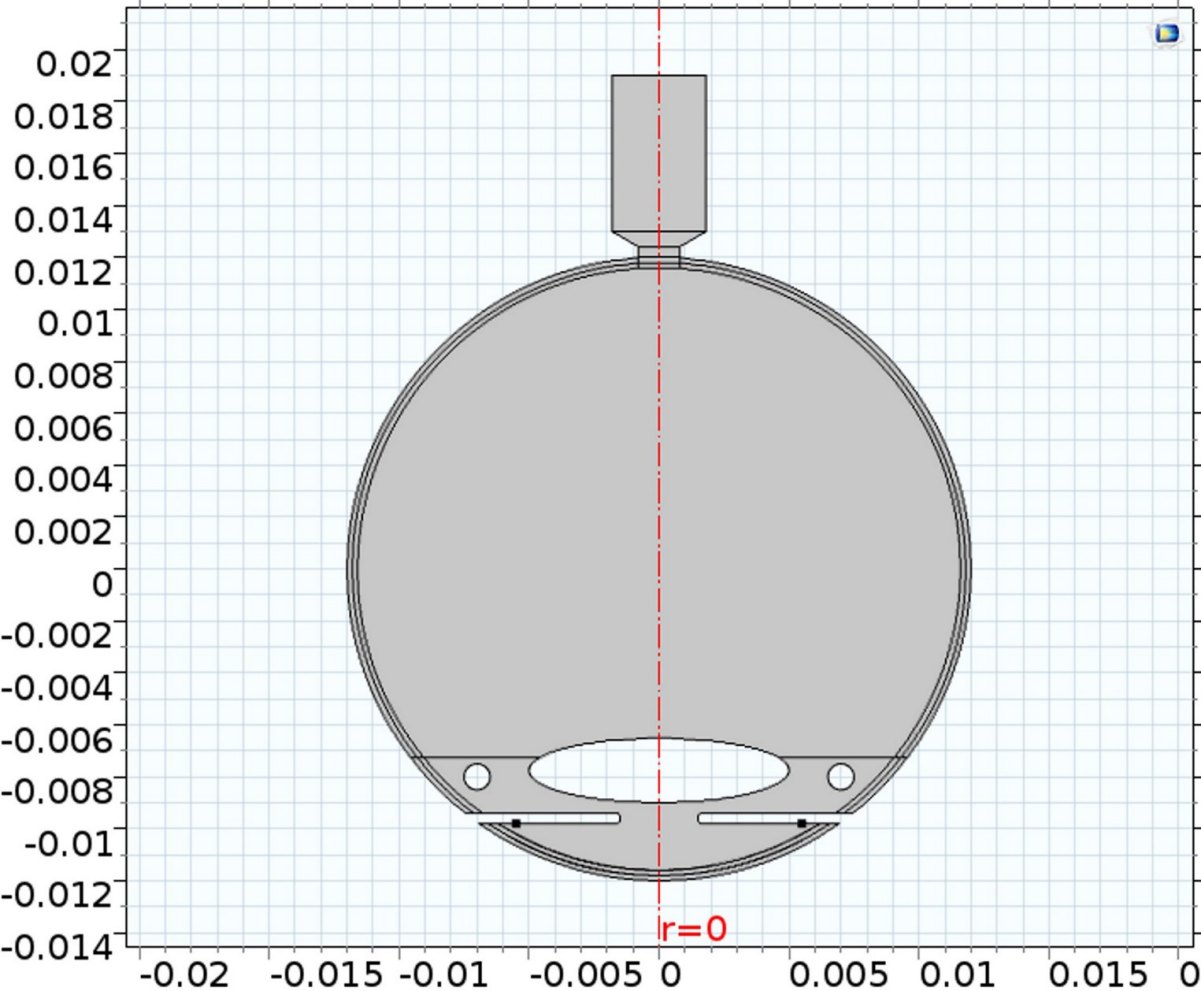
Two dimensional geometry of the eye (see measurements S1 Table; scale in metres).

SI units are employed in the computational model for all analyses, but to make the presentation of information more familiar we report in units more often used in eye physiology, as deemed appropriate (for examples, we report IOP in mmHg, and the aqueous production rate microlitres/min). The 2D geometry cross-section of the model normal eye is shown in Fig 3. Further geometric details of the model normal eye are given in (S1 Table).

### Model parameter estimates and initial check on model

A key parameter for the fluid outflow model is the rate of aqueous production. This flow rate has been experimentally estimated for the pressure-dependent outflow model described in [34]. Here we adopt an initial aqueous production rate of 6.32 microlitres a minute initially. For the parameters in Eq (4), we assume for the initial model that $C_{T}^{SL}=1.0$ where $C_{T}^{SL}$ (μL/min/mmHg) is the total hydraulic conductivity across outflow surfaces at all anatomic outflow structures of the eye, α=0.075(mmHg^-1^) is a parameter describing the decrease in outflow facility with increasing IOP, while $p_{T}=3$ mm Hg (i.e. 400 Pa) and is constant, ‘no-flow’ pressure at each outflow structure.

The retina and optic nerve is an extension of the brain, so it has similarities to gray and white matter of the brain. Due to the limited available data for the retina and optic nerve flow properties, we also consider data on the hydraulic conductivity of the gray and white neural tissues for the mammalian brain, preferably with measurements made *in vivo*. The properties of the vitreous humor are of great importance in estimating pressure gradients throughout the vitreous (and at the back of the eye close to the optic disc), so we also consider this experimental data. Finally, we also report on some parameters used in some previous flow models for the eye. All our reference data on hydraulic conductivities are summarized in (S2 Table).

The initial porous media model parameters for our model are reported in column 1 of (S3 Table). This initial model is designed so that it should return exactly the same predictions as the ODE model reported in [34]. This provides a valuable check that the 3D porous media model and the ODE model developed in [34]—both models initially agree.

Our initial model has an aqueous production rate of 6.32 microlitres per minute and parameter values: α equals 0.075 (mmHg^-1^), $p_{T}$ equals 3 mmHg, $p_{back}$ equals zero and $C_{T}^{SL}$ equals 1.0 (μL/min/mmHg). The total hydraulic conductivity across outflow surfaces, in the porous media model is taken to be $C_{T}^{SL}=C_{pre}^{SL}+C_{ap}^{SL}$, where we have divided the hydraulic conductivity across outflow into a flow across retinal pigment epithelium (subscript ‘*pre*’ for retinal pigment epithelium), and the hydraulic conductivity outflows anteriorly (subscript ‘*ap*’ for anterior pathways)). For our initial model (which returns exactly the same predictions as the ODE model reported in [34]), we attribute 50% of the hydraulic conductivity outflow to surfaces at anterior pathways and 50% for the surface hydraulic conductivity determining fluid flow across the retinal pigment epithelium, where it is almost entirely resorbed into the choroidal capillaries. That is, $C_{ap}^{SL}$ is the sum of hydraulic conductivities across the conventional and unconventional flow pathways, and any outflow attributed to pseudo-facility may also be lumped with the anterior pathways.

We then find this initial porous media model predicts an IOP of 15 mm Hg throughout the eye, exactly the same as the IOP predicted by ODE model reported in [34]. We can now sequentially introduce tissue hydraulic conductivities within the 3D eye model and its optic nerve, and so build our 3D flow model for a normal eye.

### Building the 3-D flow model for the normal eye

We now explore the effects of introducing distributed flow resistances into the initial 3D model (which are identified in (S2 Table)) transforming the initial 3D model into the 3D flow model for the normal human eye in the upright position. We add the flow resistances sequentially to the initial 3D model, and report the effect they have on the predicted IOP, as shown in column 2 of (S3 Table). We note that for a 3D model of fluid flow for the eye, fluid pressure may be spatially variable throughout the eye. Consequently in the following, unless otherwise specified, IOP is now defined to refer to the fluid pressure in the anterior chamber (where it is normally measured clinically). For all the models reported here, the anterior and posterior chambers are given a very large hydraulic conductivity, so the IOP in the anterior chamber is effectively constant, and this fluid pressure differs from the IOP in the posterior chamber by only a tiny fraction of a mmHg.

While keeping aqueous production and all other model parameters constant, first we give retinal tissue a flow resistance (i.e. we increase the flow resistance from a vanishingly small quantity to a more plausible finite value, by decreasing the hydraulic conductivity). Assuming aqueous production is constant, we find the IOP always increases because the retina now represents a flow resistance to the outflow pathway across the retinal pigment epithelium. How much the IOP rises depends on the magnitude of the decrease in hydraulic conductivity of the retinal tissue (see second row in (S3 Table)).

There have been at least two attempts to directly measure the hydraulic conductivity of retinal tissue, but it is difficult to do this experimentally because retinal tissue is so fragile. For this reason, investigators have peeled the retinal tissue away from the pigmented epithelium and placed it on a ‘support membrane’ for testing [40, 41].

Antcliff et al. reports human retinal hydraulic conductivity to be $k_{ret}=5\times 10^{-14}$(m^2^/Pa-s), while Fatt and Shantinath reported a rabbit retinal hydraulic conductivity of $k_{ret}=9.4\times 10^{-12}$, a difference of almost 200 times! We note that Antcliff et al. claim that their method cannot be compared to the data reported by Fatt and Shantinath, because it is not possible to transform their data into the units used by Antcliff et al., but that is not true. In fact Fatt and Shantinath have used a technically correct analysis of their data that takes into account the permeability of the support membrane on which the retina is mounted due to the retina’s propensity to tear (we note that the membrane used by Fatt and Shantinath had larger pore opening than the membrane by used by Antcliff et al., yet the support membrane measured permeability used by Fatt and Shantinath was still about 10% that of the retina). While the reason for this discrepancy in measured permeability is uncertain and would require further experimental testing to determine, we note that rabbit and human retina both have inner and outer plexiform layers, which Antcliff et al. claim are the retinal layers primarily responsible for the low measured permeability (we mention in passing that excluding these layers leads to a permeability more like than reported by Fatt and Shantinath).

Given this uncertainty we sought additional guidance. It is known that gray matter of the mammalian brain (which is similar to retinal tissue) has an experimentally estimated hydraulic conductivity of about $2.1\times 10^{-12}$, while an estimated hydraulic conductivity for gray matter of $5.0\times 10^{-12}$ has been employed in numerical models previously (S2 Table). Because $5.0\times 10^{-12}$ sits at the lower end of the range found experimentally by Fatt and Shantinath, and also because this estimate sits about midway between the hydraulic conductivity for gray matter and the mean value for rabbit retina measured by Fatt and Shantinath, we adopt $5.0\times 10^{-12}$ (m^2^/Pa-s), as our best initial estimate for human retinal hydraulic conductivity.

When we introduce a retinal hydraulic conductivity of $5.0\times 10^{-12}$ (m^2^/Pa-s) into the flow model with constant aqueous production, we find the IOP increases by just 0.006 mmHg (see S3 Table). We also find that this leads to a tiny pressure drop of just 0.8 Pa across the retina itself. However using Antcliff et al.’s mean estimate for retinal permeability ($k_{ret}=5\times 10^{-14}$(m^2^/Pa-s)) the pressure drop is across the retina is about 143 Pa (1.07 mmHg).

When the retina detaches and any breaks are subsequently closed surgically, or the retina detaches intact (perhaps due to an exudative accumulation of subretinal fluid), a retinal bleb forms. If the cause of the exudative process is addressed, or upon surgical closure of the retinal break, fluid in the bleb is then resorbed across the retinal pigmented epithelium, and the fluid pressure drop across the retina will return the retina to its original position on the pigmented epithelium (see for examples of this happening in reports by [42–44]). Given the tamponade buoyancy pressure exerted on a rhegmatogenous retina by (standard) silicone oil is about 20 to 40 Pascals (the pressure depends on the depth of oil measured in the direction of gravity), then the pressure drop across the retina estimated using Fatt and Shantinath’s measured hydraulic conductivity for the rabbit of less than 1 Pa is probably too small to return the retina to its original position. We therefore adopt Antcliff et al.’s mean estimate for human retinal tissue as our final best estimate for the retinal hydraulic conductivity (i.e. $k_{ret}=5\times 10^{-14}$(m^2^/Pa-s)), which has an estimated pressure drop across an intact retina under normal conditions of greater than 100 Pa. Of course, these estimates can be modified when new high quality data becomes available.

Next we introduce the vitreous humor into the porous media model (S3 Table). The vitreous hydraulic conductivity ($k_{vit}$) was estimated by Fatt and Weissman to be $k_{vit}=3\times 10^{-12}$ (m^2^/Pa-s) for rabbit vitreous and $k_{vit}=6\times 10^{-12}$ for bovine vitreous [45]. Using a more theoretically sound method for estimating hydraulic conductivity [46] (see Xu et al.’s comment on Fatt’s analysis), based on testing 14 vitreous specimens, Xu et al. reports hydraulic conductivity of bovine vitreous to be $k_{vit}=8.4\times 10^{-11}$$\pm 4.5\times 10^{-11}$ [47]. Consequently, while recognising the hydraulic conductivity estimates for bovine vitreous have reasonably wide error bounds, we have first chosen to use Xu et al.’s mean experimentally measured hydraulic conductivity. With this hydraulic conductivity and a constant rate of aqueous production, we find the IOP increases by about 0.05 mmHg. We note that if we set $k_{vit}=1.0\times 10^{-11}$, we find the IOP increases by about 0.41 mmHg (18.11 mmHg– 17.70 mmHg), suggesting the hydraulic conductivity estimate for the vitreous reported in Fatt and Weissman (which is about half this value, which would approximately double the IOP increase to 0.82 mmHg) is also plausible, but possibly too low for normal vitreous. Interestingly, using Xu et al.’s vitreous estimate there is now a small pressure drop between the anterior chamber and retina at the back of the eye of about 0.14 mmHg, representing about a 1% decrease in ocular pressure. Of course this vitreal permeability estimate can change if new high quality experimental data becomes available.

Next we introduce a ‘leakage’ of fluid through the optic nerve head, driven by the TLPG (see row 4 in (S3 Table)). That such a flow exists is demonstrated by the movement of small molecules through the interstitial spaces between retinal axons [27, 28]. Axoplasmic fluid flow has also been proposed by [29]. We note that the ‘hydraulic conductivity’ of the optic nerve head tissues and optic nerve tissue is the average hydraulic conductivity for each tissue, which includes both interstitial transport through the extracellular matrix and intracellular fluid flow along the axons. We now calibrate the model aqueous production so that IOP at the front of the eye is 15 mmHg, and examine the hydraulic conductivity of the optic nerve head and optic nerve more closely.

Unfortunately, there are no experimental hydraulic conductivity measurements for the optic nerve tissue available in the literature. However it is reasonable to expect that the hydraulic conductivity of the optic nerve ($k_{ON}$) would be similar to that of the white matter nerve tracts of the brain. Indeed, the optic nerve turns into the ‘optic tract’ and later the ‘optic radiation’ within the brain. Based on a mean hydraulic conductivity of white matter measured in the brain for the cat and rat species (S1 Table), the hydraulic conductivity of the (myelinated) optic nerve is with some confidence, taken to be about $k_{ON}=1.6\times 10^{-11}$(m^2^/Pa-s). Now given the known geometry of the optic nerve head, once the optic nerve hydraulic conductivity had been estimated with reasonably high confidence, it is then possible to estimate the hydraulic conductivities for $k_{ONPL}$, $k_{ONHLC}$ and $k_{ONRL}$ based on the detailed experimental observations made on dogs as reported in [30] and [37]. Specifically, Morgan et al. reports that 85% of the pressure drop from the IOP at the optic disc to the retrolaminar neural tissue occurs across the 400 microns or so of the laminar cribrosa [30]. Morgan et al. also give a normalized pressure curve with depth, extending from the superficial layer of the optic nerve head through to the retrolaminar optic nerve tissues (see Fig 6 in [37]). Given the hydraulic conductivity of the optic nerve, one can then adopt the strategy of adjusting the hydraulic conductivities in our model eye so that $k_{ONHPL}$, $k_{ONHLC}$ and $k_{ONRL}$ relative to $k_{ON}$ lead to a match with the depth-dependent pressure data reported in [37].

After temporarily setting the pressure on the (post-laminar cribrosa) surface of the optic nerve to 5 mmHg, a value consistent with Fig 7 in [37], we do a ‘trial and error’ exploration of hydraulic conductivities so as to match the Morgan et al. pressure profiles through the optic nerve head. We find the hydraulic conductivity of retrolaminar tissue ($k_{ONRL}$) is about $k_{ONRL}=9.1\times 10^{-13}$, the hydraulic conductivity of the laminar cribrosa in optic nerve head is $k_{ONHLC}=7.7\times 10^{-14}$ and for the superficial and prelaminar regions of the optic nerve head we estimate a hydraulic conductivity of $k_{ONHPL}=8.3\times 10^{-13}$.

Interestingly we observe that the hydraulic conductivity for the laminar cribrosa ($k_{ONHLC}$) is about the same as that for the retina. This low hydraulic conductivity region through the optic nerve head relative to the myelinated optic nerve itself ($k_{ON}/k_{ONHLC}\approx 200$) makes practical sense from the viewpoint of reducing the fluid flow rate through the optic nerve head (see later discussion and Eq (6) on drag forces induced by fluid flow along axons).

We also observe that the low hydraulic conductivity tissues in the region of the optic nerve head are coincident with the reported high density of (light, medium and heavy) neurofilaments (and heavy phosphorylated neurofilaments) in this anatomical location [39]. Indeed, the predicted low hydraulic conductivity at the superficial layer in the optic nerve head is also consistent with the presence of high density of (light, medium and heavy) neurofilaments and heavy phosphorylated neurofilaments [39]. That there are identifiable increases in intracellular neurofilaments concentrations in the optic nerve head provides some evidence for there being a physical basis for the lower hydraulic conductivity that we have estimated above in this region.

Morgan et al. has also found by experiment that there is a ‘floor pressure’ of about 2.6 to 4.4 mmHg in the CSF pressure in the sub-arachnoid space surrounding the optic nerve, which is probably set by the ‘orbital pressure’ behind the eye [25, 26]. For example, they report [37]:

Retrolaminar tissue pressure (5 mm Hg) did not vary when cerebrospinal fluid pressure was reduced from 0 mm Hg to—5 mm Hg and then increased to 2 mm Hg. However, it moved with cerebrospinal fluid pressure when cerebrospinal fluid pressure was increased above 5 mm Hg.

For the hydraulic conductivities mentioned in the previous paragraphs, and assuming that the average pressure in the optic nerve is about 4.0 mmHg when the CSF pressure is 0 mmHg (see for examples Fig 7 in [30], and Figs 7 and 8 in [37]), we can then calibrate the optic nerve hydraulic conductivity coefficient across the pia mater ($h_{ON}$). Employing the Robin boundary condition, we find that $h_{ON}=1.5\times 10^{-11}$ (m/Pa-s), where

$$v_{dON}=h_{ON}(p-p_{CSF})$$ (5)

and $v_{dON}$ is the Darcy velocity as fluid moves across the pia mater and associated tissues from the optic nerve into the subarachnoid space, and $p_{CSF}$ is the pressure in the cerebrospinal fluid surrounding the optic nerve within the subarachnoid space. This value of $h_{ON}$ results in a retrolaminar pressure of 3.5 mmHg in the optic nerve when the subarachnoid CSF pressure is zero, compared to the measured retrolaminar pressure of 3.7 ± 0.2 mmHg reported in [37]. It is not known if $h_{ON}$ varies along the optic nerve, but in the absence of data to the contrary we assume it to be constant.

We note that the pia mater is 25 microns thick in the dog, but the rapid pressure change occurred over the first 50 microns to 100 microns of tissue (including the pia mater) [37], so $h_{ON}$ hydraulic conductivity refers to the pia mater and nearby tissues (which presumably comprises part of the ‘glia limitans externa’—see Fig 3 in [48]). Morgan et al. also report that for an IOP of 15 mmHg (measured at the back of the eye) and a CSF pressure of 0 mmHg, the normal TLPG for dogs was 23 mmHg/mm [37]. Our flow model for the normal human eye predicts a TLPG of 20 mmHg/mm (i.e. (14.0 mmHg– 6.5 mmHg)/0.38 mm), which given the uncertainties, we deem to be a good model fit to the data.

Based on the work of Moller, Morgan et al. observes that the orbital pressure for humans has been measured at 3 mmHg [37, 49]. So now we introduce a 3 mmHg ‘floor pressure’ into our model for the normal human eye, and then calibrate the model aqueous production again so that IOP at the front of the eye is 15 mmHg.

For a subarachnoid CSF pressure of 3 mmHg, we find the fluid pressure in the optic nerve (about 4 mm along from the optic nerve head) is about 5.7 mmHg, similar to the retrolaminar pressure shown in Fig 7 of [30] at a subarachnoid CSF pressure of 3 mmHg. If we now keep all other parameters unchanged and increase the subarachnoid CSF pressure to 20 mmHg, compared to Fig 7 in [30], we find that our model predicted slope for the change in retrolaminar pressure with respect to change in subarachnoid pressure probably represents a lower bound on the slope for the data shown Fig 7 in [30]. Given the uncertainties (e.g. IOP on data shown in Fig 7 in [30]), we deem our model prediction to be a reasonably good fit to Morgan et al.’s data. We again calibrate the model aqueous production so that IOP at the front of the eye is 15 mmHg. We now find the aqueous production rate is 6.14 microlitres/min. Our model calibration is now complete.

**Fig 4:**
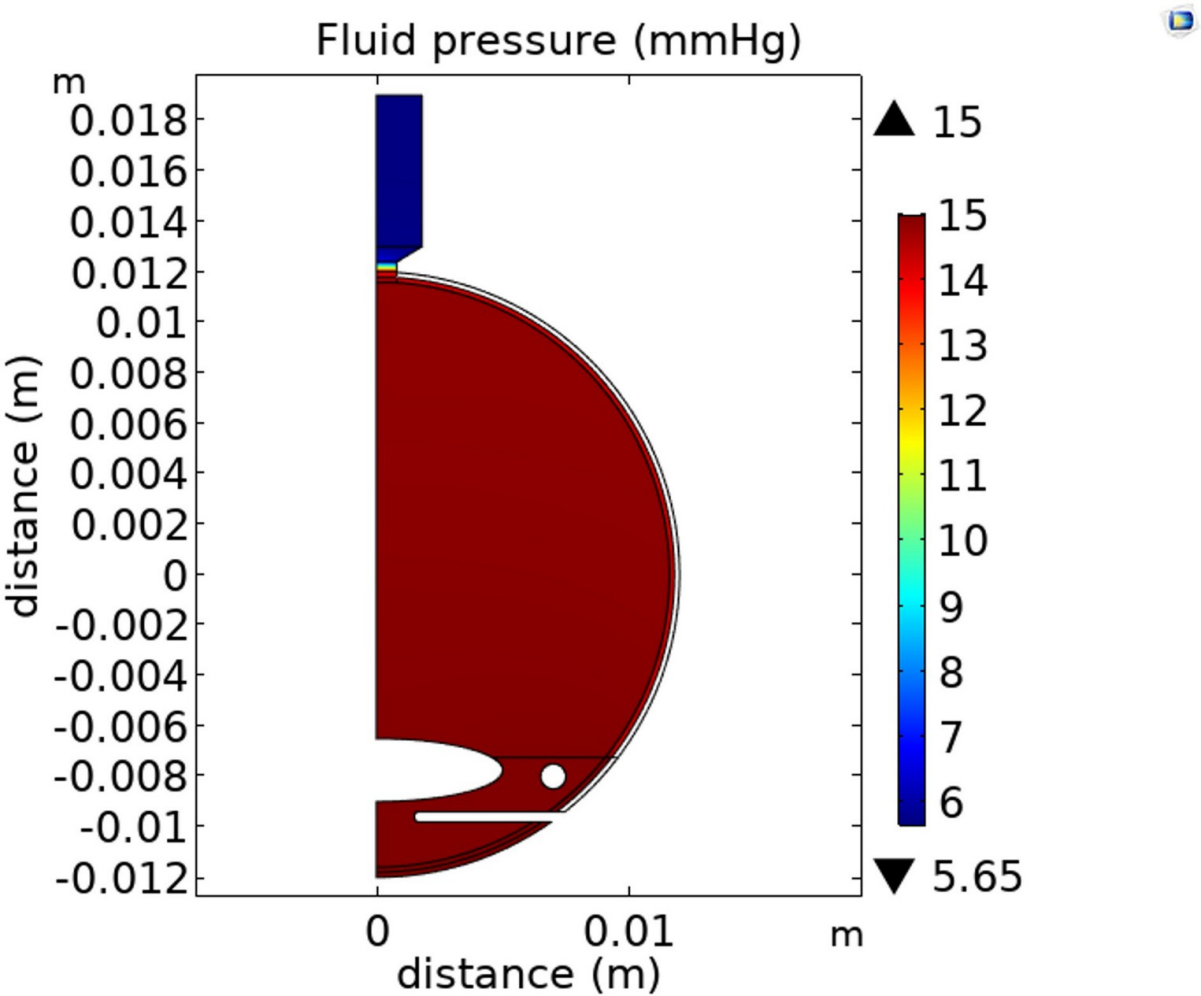
Fluid pressure (mmHg) distribution on an axisymmetric cross-section through the eye predicted by the model (scale metres).

We now refer to this calibrated base case model for the normal human eye flow model as ‘the model’ in this paper. All the parameters in the model are summarized in Table 1. The two dimensional cross-section of the pressure distribution throughout the eye is shown in Fig 4.

**Table 1:** Model parameters for normal human eye in upright position.

Model parameters for normal human eye flow model		
Parameter	Value	Units
Aqueous Production Rate	6.14	microlitres/min
$C_{T}^{SL}$	1.0	microlitres/mmHg/min
$C_{PRE}^{SL}$	0.5	microlitres/mmHg/min
$C_{AP}^{SL}$	0.5	microlitres/mmHg/min
*α*	0.075	1/mmHg
$p_{T}$	3	mmHg
$p_{back}$	0	mmHg
$p_{CSF}$	3	mmHg
$k_{ac}$	$1.0\times 10^{-5}$	m^2^/Pa-s
$k_{vit}$	$8.4\times 10^{-11}$	m^2^/Pa-s
$k_{ret}$	$5.0\times 10^{-14}$	m^2^/Pa-s
$k_{ON}$	$1.6\times 10^{-11}$	m^2^/Pa-s
$k_{ONRL}$	$9.1\times 10^{-13}$	m^2^/Pa-s
$k_{ONLC}$	$7.7\times 10^{-14}$	m^2^/Pa-s
$k_{ONPL}$	$8.3\times 10^{-13}$	m^2^/Pa-s
$h_{ON}$	$1.5\times 10^{-11}$	m/Pa-s

We see from Fig 4 for example, that at an IOP of 15 mmHg, the model predicts the pressure drop between the front and the back of the vitreous is 0.15 mmHg. This pressure drop is caused by a body force (i.e. drag force) distributed throughout the vitreous humor, which would cause the vitreous to ‘accelerate away’ were this force not resisted. The resistance is provided by either the collagen network transferring a tensile stress towards the lens, or the hyaluronan transferring a compressive stress towards the retina, or some combination of the two. Compressive stress transferred to the retina would also help hold the retina against the retinal pigment epithelium (maximum pressure drop to the posterior vitreous of about 0.15 mmHg), and together with a pressure drop of about 1.05 mmHg across the retina itself, this leads to an estimated maximum pressure holding the retina in place against the retinal pigment epithelium to be about 1.2 mmHg (i.e. 160 Pa).

There is a rapid pressure drop across the optic nerve head between the eye and the optic nerve (see Fig 4). For the coordinates (0, 0.016) in Fig 4, we also see the fluid pressure in the optic nerve predicted by the model is 5.7 mmHg.

**Fig 5:**
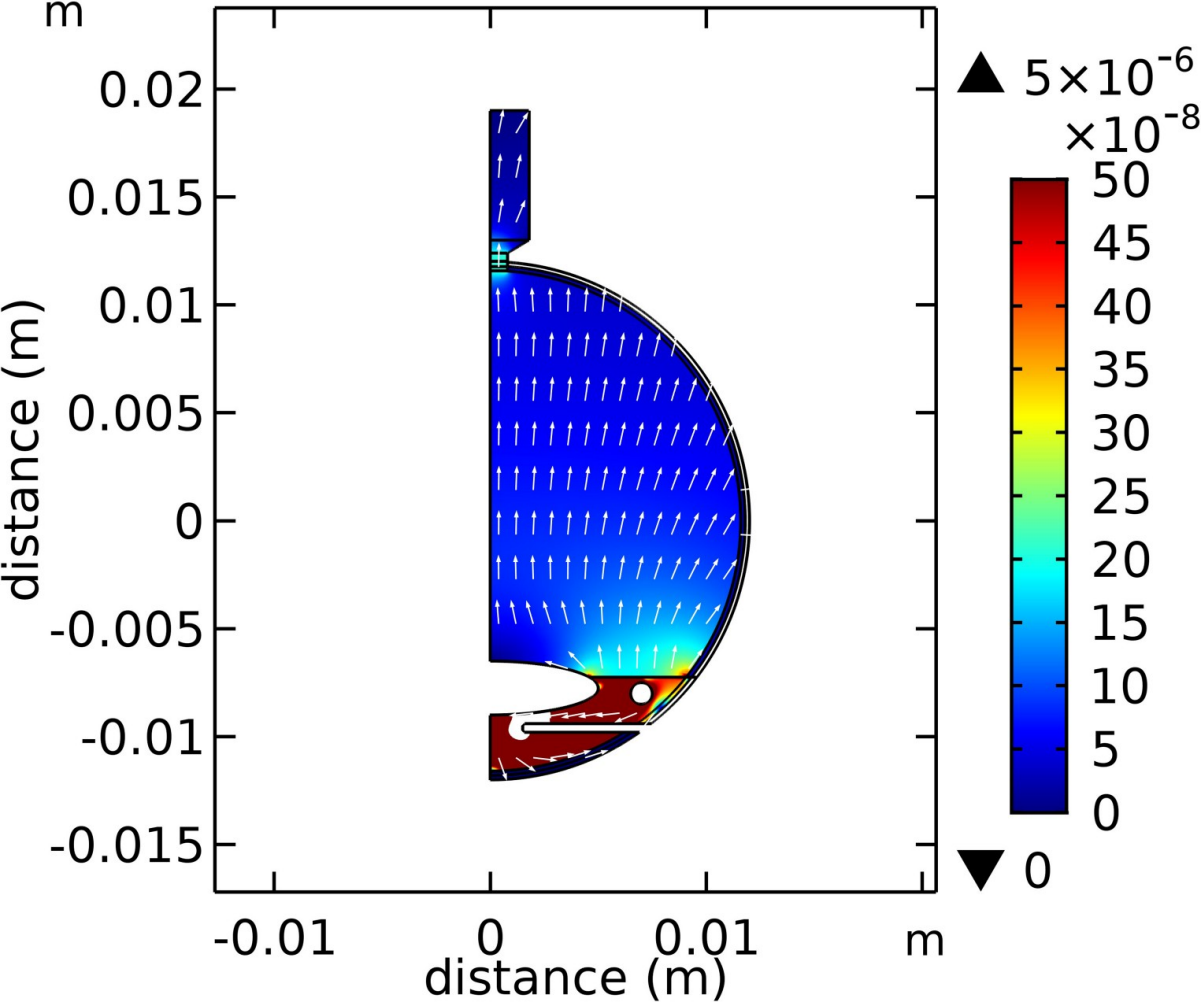
Darcy velocity distribution (m/s) on an axisymmetric cross-section through the eye predicted by the model (scale m). Notes: (i) arrow plot is normalise to one, so arrows only show flow direction, not the velocity magnitude and (ii) velocity magnitudes are indicated by the colour scale and (ii) velocity magnitudes larger than 5.0×10^−7^ (m/s) are not shown for clarity (these velocities occur in a small region where the flow paths become restricted between iris and lens).

Fig 5 shows the magnitude (colour scale) and direction (arrows) of the fluid velocity field throughout the eye. Some general observations are that the fluid velocity is much larger in the anterior and posterior chambers than in the vitreous chamber. In the vitreous chamber fluid velocities are typically about 0.1 micron/s, while in the anterior chamber they are around five times larger, except where there is a flow restriction between the iris and lens, where the fluid velocity may be a few microns per second. We see that the fluid velocity increases in the optic nerve head relative to that in the vitreous chamber, as fluid flows from the eye into the optic nerve. The fluid flow rate through the optic nerve head is predicted to be (0.0256/6.14 equals) 0.42% of the fluid production rate at the ciliary body. The fluid flow through the anterior pathways is calculated to be (3.16/6.14 equals) 51.5% of the fluid production, while the remaining 48.1% of the fluid production flows across the retinal pigment epithelium.

It is of some interest to understand the sensitivity of the IOP to parameter changes in the model. For example, what is the change in IOP with aqueous production rate? The computed sensitivities are shown in Table 2. We see that all parameters in the model can have a very significant effect on IOP, while not surprisingly the parameters associated with the optic nerve have little effect on IOP.

**Table 2:** Sensitivity of IOP for the model.

Parameter sensitivity model BC2			
Parameter	Fractional change	Fractional change in IOP	Sensitivity
Aqueous production rate	+10%	+13.3%	133%
$C_{T}^{SL}$	+10%	-10.3%	-103%
α	+10%	+8.1%	81%
$p_{T}$	+10%	+4.6%	46%
$p_{back}$/15mmHg	+10%	+10%	100%
$p_{CSF}$	+10%	0.3%	0.03%
$k_{ONLC}$	+10%	-0.4%	-0.04%

Also of interest is the sensitivity of changing the fraction of outflow through the anterior pathways versus across the retinal pigment epithelium, while keeping the total hydraulic conductivity across the outflow surfaces constant? The results for the model are shown in Table 3. We see that the IOP is not very sensitive to changes in the fractional outflow attributed to anterior and retinal pigment epithelium pathways. For example, if the fractional outflow estimates are somewhat inaccurate in their location (say ± 10%), the effect on the IOP is minimal (less than 1%).

**Table 3:** Change in IOP with change in fraction of outflow anteriorly or across retinal pigment epithelium for the model.

IOP predicted by the model with change in fractional outflow facility for $C_{AP}^{SL}$ and $C_{PRE}^{SL}$							
Anterior fraction	0.5	0.45	0.4	0.35	0.55	0.6	0.65
Retinal pigment epithelium fraction	0.5	0.55	0.6	0.65	0.45	0.4	0.35
IOP mmHg	15	15.12	15.26	15.41	14.89	14.79	14.70
Sensitivity of IOP to outflow location		8%			-6.6%		

## Results

Having described and calibrated ‘the model’ for the normal human eye in the upright position, we can now explore the implications of this model in more detail.

### Predicted translaminar pressure gradient for the normal human eye

Given the translaminar pressure gradient is crucially important to the initiation and progression of glaucoma we ask: what is the predicted translaminar pressure gradient for the normal human eye in the upright posture?

**Fig 6:**
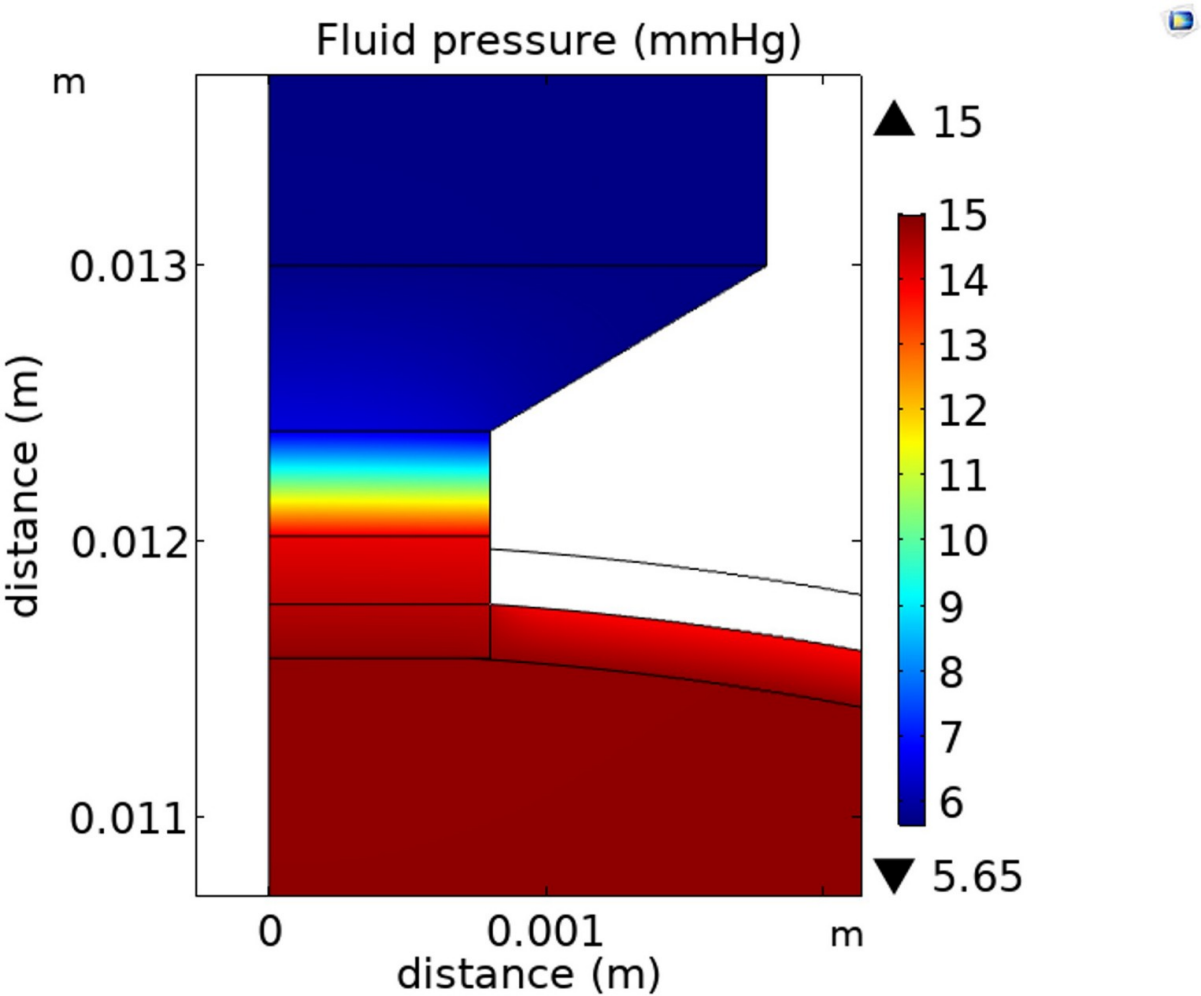
Axisymmetric fluid pressure distribution (mmHg) through a cross-section of the optic nerve head predicted by the model (scale metres).

**Fig 7:**
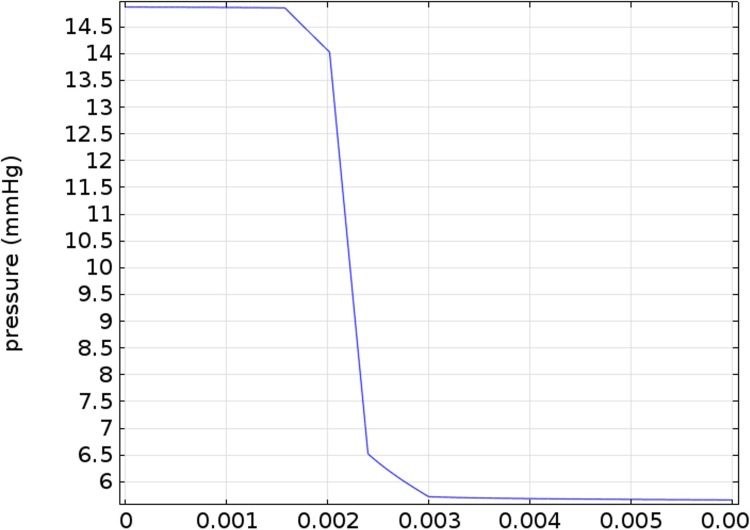
Translaminar pressure profile showing translaminar pressure gradient along the axis of symmetry predicted by the model.

At an IOP of 15 mmHg, the model predicts the pressure drop between the front and the back of the eye is 0.15 mmHg, and the midline fluid pressure in the myelinated optic nerve is close to 5.7 mmHg for a 3 mmHg orbital pressure (see Fig 6). The total pressure drop from vitreous to the myelinated optic nerve of 14.8 mmHg– 5.7 mmHg = 9.1 mmHg (see Figs 6 and 7). The midline (laminar cribrosa) translaminar pressure drop is 14.0 mmHg– 6.5 mmHg = 7.5 mmHg (i.e. about 82% of the total pressure drop occurs across the laminar cribrosa (compared to Morgan et al. who reports 85% of the total pressure across the laminar cribrosa [37])), which gives a predicted mean TLPG of 7.5 mmHg/0.38 mm = 19.7 mmHg/mm in the normal human eye (see Fig 7).

Note that normally there is about a 0.15 mmHg pressure drop from the front to the back of the eye, so if the vitreous humor is removed and all other parameters remain unchanged, the IOP in the eye becomes constant, though the IOP drops from 15 mmHg to 14.95 mmHg. In this case the TLPG will slightly decrease from 19.7 mmHg/mm to (14.0 mmHg—6.6 mmHg)/0.38 mm = 19.5 mmHg/mm. In other words, practically there is no significant change in the TLPG upon complete removal of the vitreous humor.

#### Does the TLPG change soon after assuming the supine position from a sitting position?

It is found experimentally that the cranial cerebrospinal fluid pressure (at the level of the tragus of the ear) is slightly negative in the upright position (at about minus 1.8 mmHg), but this increases to about plus 11 mmHg in the supine position (see for example Table 1 in [50]). Because the cerebrospinal fluid pressure increase is normally transferred from the lateral ventricles to the CSF in the subarachnoid space surrounding the optic nerve [30], in the supine position, the subarachnoid CSF pressure should rise to approximately 9 mmHg (allowing for a slight pressure gradient between the CSF pressure in the subarachnoid space around the optic nerve and the measured spinal cerebrospinal fluid pressure to enable fluid flow). We note that prior to lateral canthotomy the mean CSF pressure in the subarachnoid space of the prone, anaesthetized dog was found to be 8.7 ± 2.7 mmHg [37]), which closely approximates our assumed 9 mmHg increase.

It is known that the episcleral venous pressure also increases by somewhere between 1.5 mmHg and 5 mmHg, shortly after assuming the supine position from a sitting position [51–54], and that this increase in episcleral venous pressure is accompanied by a rise in IOP of approximately similar magnitude [53, 55]. Similar postural changes in IOP are observed in primates [56], while a one to one correspondence was found between rise in episcleral venous pressure and rise in IOP with head tilt in the rabbit [57].

The pressure increase in the eye caused by a change in postural position from sitting to supine is presumably driven by an increase in venous pressure around the eyeball. As this increased venous pressure is generalized, it causes an ‘all-round’ increase in hydrostatic pressure within the eye. This hydrostatic pressure increase within the eye does not drive fluid outflow from the eye, as the change in hydrostatic component of pressure inside the eye is the same as the change in hydrostatic component of pressure immediately outside the eye, along the drainage pathways. Because there is no gradient in pressure, a hydrostatic increase cannot drive fluid outflow from the eye. For this reason, hydrostatic pressure may be thought of as a ‘background pressure’, which simply shifts the baseline for fluid ‘driving pressures’ up or down.

The model assumes that the baseline background pressure is normally zero in the upright posture, which presumably corresponds physiologically to the normally zero (i.e. atmospheric) pressure in the jugular veins along the upper neck [58]. Upon adopting the supine position, for the normal cardiovascular system the pressure along the entire jugular veins in humans increase by several centimetres of water, with only a small pressure gradient along the jugular veins towards the heart (see for example Fig 2 in [59]). We note that this background pressure can be taken into account in our 3D flow model by simply subtracting the background pressure ($p_{back}$) from the total pressure (p) to find the ‘driving pressure’ (which ‘drives’ fluid outflow from the eye), as shown in equation (4).

From this we may assume that upon attaining the supine position from a sitting position, the backpressure pressure at the eye increases from zero to say 3.0 mmHg, and the pressure in the CSF in the subarachnoid space around the optic nerve increases from 3 mmHg to about 9 mmHg. Incorporating these values into our 3D flow model of the eye leads to the prediction that the total pressure drop from IOP at the back of the eye and the optic nerve is 17.3 mmHg– 11.7 mmHg = 5.6 mmHg, while the TPLG becomes (5.6 mmHg)/0.38 mm = 14.7 mmHg/mm. The predicted TLPG in the upright position of 19.7 mmHg/mm compares with the predicted TLPG in the supine position of 14.7 mmHg/mm, which represents about a 25% reduction on the normal TLPG in the upright position.

Clearly this reduction in TLPG in the supine position would be clinically significant for those with borderline glaucomatous neuropathy in the upright position. This suggests the rate of progression of glaucoma neuropathy may be related to the fractions of time spent in both positions. This leads us to consider the nocturnal TLPG.

### Predicted translaminar pressure gradient for the nocturnal eye

Does the TLPG change while sleeping in the supine position? To answer this, we need to estimate nocturnal IOP and nocturnal CSF pressure.

First we consider nocturnal IOP. For humans there is reported to be a significant circadian variation of IOP, with peak pressure generally occurring at night in younger adults (normally about 2 mmHg higher nocturnally than daytime) [55, 60], though some studies on older adults show peak pressure during the day (i.e. about 0.5 mmHg less nocturnally than daytime peak) [52]. Interestingly, telemetry data from primates averaged over a two hour window shows a weak circadian rhythm, but confusingly this rhythm changed significantly from day to day [61]. The potential causes of this circadian sinusoidal-like pressure variation are numerous. To limit the number of possibilities, we first focus on the data for older adults (mean age 59 years) reported in the paper by Nau et al., which is one the few papers to make detailed measurements of aqueous production during the day as well as at night [62].

To model the results of Nau et al., we first need to adjust our normal adult 3D eye model to a lower upright daytime IOP (reported mean IOP = 13.9 mmHg [62]). We do this, starting with the model and reducing the rate aqueous production by 6% i.e. (6.14 microlitres times 0.94) to 5.78 μL/min. The model’s anterior pathways outflow facility is 0.23 at 19 mmHg IOP, while the measured mean outflow facility is 0.23 ± 0.06 while using a Schiotz tonometer with a 5.5 gram weight and a 4 minute tracing [62]. The model outflow through anterior pathways is 2.97 μL/min, while the measured mean anterior outflow is 2.48 ± 0.96 μL/min [62]. Now we simulate the ‘night-time eye’ by: (i) reducing total aqueous production by 1.0 μL/min × 2 (anterior and posterior pathways) = 2.0 μL/min of the original daytime production (i.e. to 65% of 5.78 μL/min), which compares to a measured 51% reduction (of 2.48 ± 0.96 minus 1.27 ± 0.63 μL/min) [62], (ii) reducing anterior pathways outflow by reducing $C_{ap}^{SL}$ from 0.5 to 0.325, which reduces anterior pathway outflow by about 1.0 μL/min from its original 2.98 μL/min (compared to an estimated reduction in uveoscleral outflow of 0.94 ± 1.26 minus 0.07 ± 0.78 μL/min [62]), and, (iii) reducing outflow facility from 0.233 at 19.0 mmHg to 0.185 at 19.0 mmHg by increasing alpha from 0.075 to 0.10 (compared to the measured initial outflow facility of 0.23 ± 0.06 and nocturnal outflow facility of 0.20 ± 0.06 reported by [62]. When we make these adjustments to the model, we find the predicted IOP is 13.0 mmHg while the measured IOP is 13.0 mmHg [62]. We deem this to be a reasonable model fit to the human nocturnal eye given the variability in eye behaviour and the large uncertainties in the experimental eye measurements. For this ‘Nau et al. data modified model’, the nocturnal TLPG in the upright position is found to be is (12.2 mmHg– 5.9 mmHg)/0.38, which equals 16.6 mmHg/mm.

The nocturnal IOP of 13.0 mmHg is lower than the daytime IOP of 13.9 mmHg, but importantly it is observed that both daytime and night-time IOP were measured in the sitting position (as was the data reported by [52]). Clearly the sitting position removes known IOP changes associated with the supine position. For example the CSF pressure increases to about 9 mmHg in the supine position, while backpressure increases IOP around 2 mmHg to 4 mmHg. If we now assume a backpressure of 3 mmHg, and a CSF pressure of 9 mmHg, and leave all other model settings unchanged in the Nau et al. data modified model, the IOP rises from 13.0 mmHg to 16.0 mmHg. With these assumptions for the nocturnal supine position, we find the TLPG is (15.4 mmHg– 11.1 mmHg)/0.38, which equals 11.3 mmHg/mm. This predicted nocturnal TLPG of 11.3 mmHg/mm is even lower than the 14.7 mmHg/mm for the supine position daytime TLPG predicted by the model, and more than 40% lower than the TLPG predicted for the upright daytime TLPG by the model (see summary shown in Table 4).

**Table 4:** Estimated translaminar pressure gradient for different postures and time of day.

	Estimated translaminar pressure gradient				
	The model Upright (daytime)	The model with vitreous removed Upright (daytime)	The model Supine (daytime)	Nau et al. data modified model Upright (nocturnal)	Nau et al. data modified model Supine (nocturnal)
Estimated TLPG (mmHg/mm)	19.7	19.5	14.7	16.6	11.3
Normalized TLPG	100%	99%	75%	84%	57%

It is possible that there may be a circadian variation in the cranial CSF pressure, but there appears to be conflicting reports on a circadian variation in CSF production in humans [63, 64]. We also note that for Sprague-Dawley rats intracranial pressure in the ambulant condition was 7.5 mmHg to 10.9 mmHg, but the rats displayed no significant circadian cerebrospinal pressure variation (unlike IOP, which varied by 5.2 mmHg over the course of 24 hours) [65]. For the purposes of our best estimate model, we suppose there is no circadian variation in CSF pressure. In addition, it is possible that there may be a circadian variation in central venous pressure. For example, there is a reported circadian variation in the central venous pressure of monkeys. For rhesus monkeys (n = 4) there is about a 4mmHg increase in central venous pressure during the night while in the ‘chaired’ position [66], but no data is available for humans. For the purposes of our best estimate model, we suppose the backpressure on the eye arising from the supine position does not change.

### Predicted translaminar pressure gradient for the normotensive glaucomatous eye

Let us now consider a situation for a ‘borderline normotensive’ person, say with an IOP elevated to about 21 mmHg (which is sometimes adopted as the upper limit of the normal range for IOP). And rather than a positive orbital pressure of about 3 mmHg, let us now assume that for some reason there is a negative CSF pressure in the subarachnoid space surrounding the optic nerve. Perhaps this negative pressure in the upright position is due to surface roughness of subarachnoid tissues (which prevents the local orbital pressure tightly closing the subarachnoid space around the optic nerve)? If tight closure of the subarachnoid space is prevented, then a pressure connection with cranial CSF pressure will be maintained even for negative pressures. In the upright position, normal cranial pressure at the level of the tragus of the ear is about minus 1.8 mmHg [50]. Based on the known pressure reduction with increasing height in a stationary water column, the pressure at the level of the eye is probably 2 to 3 mmHg lower given the tragus is normally located a few centimetres below the mid-eye level (giving a predicted CSF pressure at the level of the eye of minus 3.8 to minus 4.8 mmHg, similar to the pressures reported for normal adults by [67]).

For most tissues, interstitial fluid pressures are slightly negative (e.g. zero to minus 4 mmHg [37]). The usual reason for the negative interstitial tissue fluid pressure is active lymphatic pumping capacity relative to the fluid flow rate from nearby fluid sources. But in the case of the optic nerve, it is the negative pressure in the negative cranial CSF pressure driving the negative pressure in the subarachnoid space around the optic nerve. For the upright position, Aperin et al. use MRI measurements to estimate CSF pressure at the tragus in 10 healthy adults (mean age 29 years), as minus 3.4 ± 1.75 mmHg (with range based on automated analysis of minus 5.2 mmHg to minus 0.7 mmHg, with 4/10 measurements less than minus 4 mmHg) [68]. Taking into account the hydrostatic pressure associated with the height of the eye above the tragus, Linden et al. estimated cranial pressure at the level of laminar cribrosa in the sitting position for NTG adults (n = 13) with mean aged 71 years, and a normal group of adults (n = 51) with mean aged 68 years, and found minus 4.9 ± 2.7 mmHg for NTG adults and minus 4.7 ± 3.6 mmHg for the normal group (see Table 3 in [67]). Given this data, let us assume the CSF pressure in the subarachnoid space around the optic nerve for the NTG group is minus 4 mmHg. What then, is the TLPG?

We first raise the IOP to 21.0 mmHg by changing alpha from 0.075 to 0.10 for the model in the upright position, and we then apply a subarachnoid CSF pressure of minus 4 mmHg. We leave all other model parameters unchanged. This results in a total pressure drop from vitreous to optic nerve of 20.9 mmHg– 1.6 mmHg = 19.3 mmHg, and a translaminar (cribrosa) pressure drop of 19.1 mmHg– 3.4 mmHg = 15.7 mmHg. This results in an estimated TLPG of (i.e. 15.7/0.38 mmHg/mm), which equals 41.3 mmHg/mm. This TLPG represents a 210% increase relative to the normal model TLPG of 19.7 mmHg/mm.

Fleischman et al. review CSF pressure in adults (as measured by lumbar puncture [69]), and report that it begins to decrease at about age 60, so that by 90 it is about 3 mm lower (11.5 mmHg age 20 to 49, versus 8.4 mmHg age 90 to 95) [70]. Based on this data we suppose an additional 3 mmHg in negative pressure for an adult of advanced years, so we apply minus 7 mmHg to the CSF in the subarachnoid space surrounding the optic nerve. For this case we find the TLPG increases to (18.8 mmHg– 1.3 mmHg)/0.38 mm), giving a (NTG model 2) predicted TLPG of 46.1 mmHg/mm.

In the context of fluid pressure gradients along peripheral nerves, there is one particularly relevant paper that attempts to quantify the effect of a fluid pressure gradient applied along the vagus nerve for the rabbit, on the (room-temperature) axoplasmic transport of radiolabelled leucine along the motor neurones within this vagus nerve [71]. Hahnenberger reports that following 17 hours of pressure testing at room temperature:

When a nerve was subjected to 20 mmHg/mm pressure fast axoplasmic flow was not altered, but at 30 mmHg/mm there was a slight but consistent inhibition, which was even more marked at 60 mmHg/mm and still more at 90 mmHg/mm.

This data provides evidence for a biological link between fluid pressure gradients along nerves and interruption of axoplasmic transport, but in addition, it provides quantitative evidence that 30 mmHg/mm is at about the threshold fluid pressure gradient that causes dysfunction in axoplasmic transport in motor neurones in the vagus nerve of the rabbit after 17 hours. Though it is not practical to test experimentally, it is probable that given chronic exposure to pressure gradients, perhaps measured over months or even years, it would be observed that somewhat smaller axoplasmic fluid pressure gradients would also lead to dysfunction of axoplasmic transport, while a shorter exposure duration (e.g. 8 hours) would lead to a somewhat larger threshold axoplasmic fluid pressure gradient. How long a pressure gradient threshold actually is in place in humans remains to be determined, but based on the findings in this paper, we now know that a TLPG of 19.7 mmHg/mm in the upright position is probably normal and safe, at least when it is combined with regular periods of sleep in the decubitus position (where the nocturnal TLPG may drop 25% to 40% and so provide relief from upright, daytime TLPGs).

In this context, it is of interest to define a ‘factor of safety’. A factor of safety is simply a number that helps one better appreciate the probability of some adverse event. For example, we note that Biewener defined a factor of safety against bone fracture as the ultimate stress a bone is capable of withstanding, divided by the ‘working stress’ for that bone (where the working stress is the maximum stress experienced in the long bones while an animal is running, hopping, jumping or galloping, as the case may be). Interestingly, Biewener found that the factor of safety against bone fracture was about 2.0 to 4.0 [72].

For our particular application, we now define a ‘factor of safety’ as the pressure gradient of 30 mmHg/mm (i.e. the threshold pressure gradient reported by [71] for acute (17 hours) interruption of axonal transport in motor neurones in the vagus nerve of the rabbit) divided by actual (or predicted) TLPG. When the factor of safety is equal to one or less than one, axonal transport is acutely compromised, and when the factor of safety is significantly greater than one axonal transport is not compromised. Interestingly, we see that for the normal eye in the upright posture, the estimated factors of safety is a little less than 1.5 rising to about 2.5 in the supine position at night (see Table 5). A time weighted average may be around 2.0, depending on lifestyle. However for the model NTG eye, the factor of safety is always less than 1.0, suggesting the development of a glaucomatous neuropathy is likely, and this becomes more likely with advancing years (see Table 5).

**Table 5:** Estimated translaminar pressure gradient and factors of safety for normal and normotensive eyes, in the upright and supine postures in the daytime and at night-time.

Estimated translaminar pressure gradient					
Model	Normal model Upright (daytime)	Normal model Supine (nocturnal)	NTG model 1 Upright (daytime)	NTG model 2 Aged adult Upright (daytime)	Threshold for short-term axoplasmic flow interruption [71]
Estimated translaminar pressure gradient (mmHg/mm)	19.7	11.3	41.3	46.1	30.0
Safety factor 30.0/TLPG	1.52	2.65	0.73	0.65	1.0

### Can an elevated translaminar pressure gradient predicted by the model explain interruption of retrograde neurofilament transport?

Neurofilaments are intermediate filaments in neurons. Neurofilament sub-units are identified based on molecular weight, designated heavy (NF-H), medium (NF-M) and light (NF-L) [73]. Heavy and medium neurofilaments bear carboxy-terminal domains that can link with each other and with other cellular structures and organelles, forming a dynamic cytoskeleton that is constantly remodelled and renewed by axonal transport of neurofilaments [74]. Neurofilaments appear to be essential for radial growth of axons during develop, for the subsequent maintenance of axon calibre, and for the normal transmission of action potentials along axons [73].

Neurofilaments are transported along microtubules by ‘slow axonal transport’ at average speeds typically between 0.3 to 3 mm/day [75]. Surprisingly, direct observation of neurofilaments have shown they actually move about two orders of magnitude faster than their average speed—the discrepancy between the two observations can be explained by neurofilaments moving intermittently, alternating long pauses with short, stochastic bouts of fast transport [76]. Shah et al. has shown that for bovine spinal cord, the velocity of fast movements have a probabilistic distribution of velocities, and the movements are bi-directional, meaning that neurofilaments are transported in both anterograde and retrograde directions, each with their own probability distribution [74].

The movement of neurofilaments has been carefully studied in the optic nerve of mice [77–79]. Based on the data of [77] and [79] and using some model estimates made in [80], Li et al. have developed a mechanistic, bidirectional, ‘stop-go’ computational model of neurofilament transport for the mouse optic nerve [76]. Li et al. successfully calibrated their model of neurofilament transport (based on the ‘time-weighted average velocity’ of axonal transport in the mouse and rat superior cervical ganglion reported in [80]), by using a time-weighted mean fast transport velocity of 0.5 μm/s in the anterograde direction, and the time-weighted mean fast transport velocity of 0.6 μm/s in the retrograde direction [76]. We shall use this time-weighted mean (fast) transport velocity of 0.6 microns/s in the retrograde direction in our analysis to follow.

It has been shown that dynein molecular motors, moving along microtubules within axons, are responsible for retrograde axonal transport [81]. The dynein motors move along with a velocity that is proportional to their pulling force. So dynein motors move along microtubules at maximum speed when unrestrained, and they generate maximum pulling force as they stall (they ‘stall’ at some load, meaning their velocity drops to zero) [82, 83]. There is an approximately linear relationship relating dynein’s velocity and dynein’s generated pulling force, the proportionality constant depending on, among other things, the ATP concentration and the number of dynein motors working together in ‘teams’ to generate the pulling force on the load [83, 84].

The load or ‘drag force’, which opposes the pulling force generated by the dynein motors, is primarily determined by the cargo that is tethered to the dynein motors. The drag force ($F_{D}$) depends on a number of factors (including size and shape of the cargo [85], obstacles in the cell cytosol [86] and the cargo’s proximity to the axonal cell membrane [87]). Nevertheless, the drag force on small objects moving through a solution can be calculated using a standardized equation, viz [85]:

$$F_{D}=fv_{r}$$ (6)

where f is known as the ‘friction factor’ and $v_{r}$ is a velocity. Crucially, the velocity $v_{r}$ in equation (6) is the relative velocity between the cargo and the surrounding fluid, not the relative velocity between cargo and the axonal cell membrane (which is stationary). If the fluid velocity inside the axon is zero, then relative velocity between cargo and fluid, and cargo and axonal cell membrane, are the same, and distinguishing between the two is then unimportant. In nearly all of the above-mentioned experiments on the behaviour of dynein motors, the experiments have been performed on axons in which the fluid is stationary within the axon (i.e. the fluid within the axons is only perturbed by local disturbances associated with moving cargos along the axon. On spatial averaging, these local fluid velocities due to disturbances sum to zero).

But for the case of the retinal ganglion cell axons passing through the region of the optic nerve head pressure gradient, there is normally a significant net fluid velocity along the axon, and this fluid velocity necessarily causes a drag force on the dynein cargos (even in the absence of any cargo movement relative to the axonal cell membrane). In the case of the TLPG, the relative velocity between the fluid moving in the anterograde direction and cargo moving in the retrograde direction is now the (vector) sum of the dynein motor velocity and the average true fluid velocity ($v_{t}$), which has been defined in Eq (3)).

If we now employ a published estimate for the time-weighted mean (fast) transport of neurofilaments in the retrograde direction as being 0.6 μm/s [80], we can estimate the average dynein motor velocity in the retrograde direction relative to the cell membrane (${\bar{v}}_{dynein}$, where the overbar denotes the average absolute dynein velocity relative to the cell membrane) as follows,

$${\bar{v}}_{dynein}=0.6\;\text{micron/s}-v_{d}/n_{f}$$ (7)

Note that when the $v_{d}/n_{f}$ is a positive quantity (positive being the anterograde transport direction), the mean dynein velocity is reduced, and when $v_{d}/n_{f}$ equals 0.6 μm/s, the mean dynein velocity relative to the cell membrane is reduced to zero. We now estimate $v_{d}/n_{f}$ for human axons as they pass through the laminar cribrosa for normal and NTG eyes.

**Fig 8:**
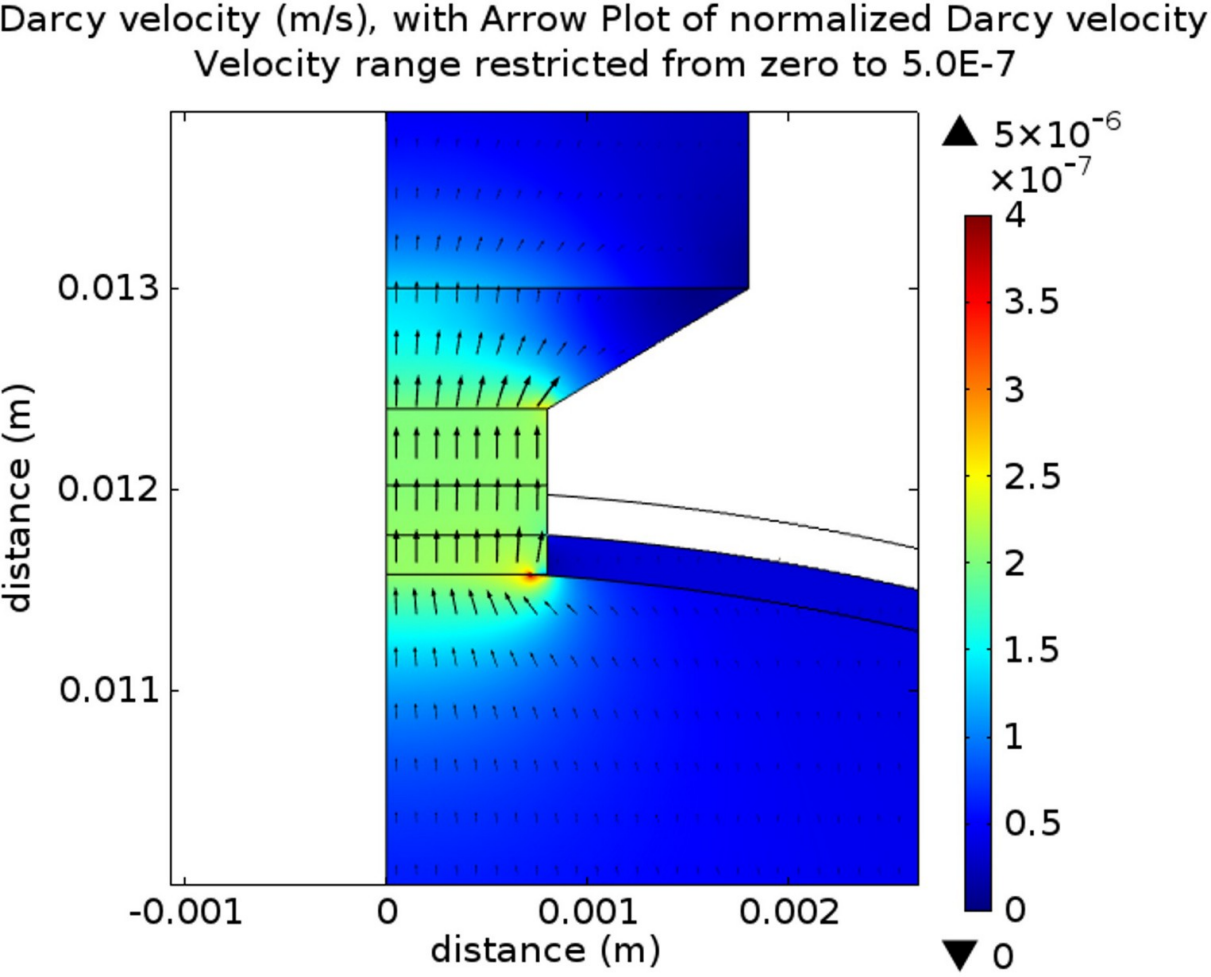
Translaminar velocity field (m/s) for the upright posture: Model for a normal eye (scale metres). Arrow length proportional to the actual Darcy Velocity, the proportionality constant being 800.

Based upon visual inspection of electron micrographs of retinal ganglion cells axons passing through the laminar cribrosa (e.g. Fig 4B in [88] or Figs 10 and 12 in [89]), the fluid porosity is estimated to be about 0.70 to 0.75, while the Darcy velocity can be estimated using our calibrated model (see Fig 8).

For the model (normal human eye, IOP = 15 mmHg in the upright posture), we estimate the maximum Darcy velocity at the along centreline of the laminar cribrosa as 0.209 μm/s, with the maximum Darcy velocity at the periphery of the laminar cribrosa as 0.216 μm/s. The velocity is about 3.3% larger at the periphery of the laminar cribrosa than along the centreline of the laminar cribrosa. The average true velocity along the centreline of the laminar cribrosa is then found using Eq (3) and porosity 0.7, as about 0.30 μm/s, while at the periphery of the laminar cribrosa we find about 0.31 μm/s.

We now assume that optic nerve damage will occur when the neurofilament network can no longer be maintained by adequate retrograde transport of neurofilaments. Clearly maintenance of the neurofilament network will be substantially compromised when the velocity of retrograde transport falls to zero i.e. at the average stall speed for dynein. That is, when

$${\bar{v}}_{dynein}=0$$ (8)

Given that retrograde neurofilament velocities form an approximately symmetrical probability distribution under no axonal flow conditions, Eq (8) implies about half the dynein motors in the probability distribution under no axonal flow would have stalled, which may be reasonably supposed to compromise maintenance of the neurofilament network in the axons. We therefore assume zero average dynein velocity is the threshold that will over a period of time compromise the neurofilament (and microtubule) network, and so axonal transport.

We can now calculate a ‘factor of safety’, defined to be 0.6 μm/s divided by the average true fluid velocity. When the factor of safety is equal to one, retrograde transport is compromised, and when it is significantly greater than one, retrograde transport is not compromised. We can also calculate the factor of safety for our model modified to represent the NTG eye in the upright position. These factors of safety are reported in Table 6. For comparison, also shown in Table 6 are the factors of safety computed previously based on the ratio of translaminar pressure gradients (see Table 5).

**Table 6:** Comparison of factors of safety against glaucomatous neuropathy based on a ratio of velocities or based on a ratio of translaminar pressure gradients.

	Factors of Safety against glaucomatous neuropathy				
	Normal eye (upright)		Normal eye (supine and nocturnal)	NTG eye model 1 (upright)	
Factor of safety	centreline	periphery	centreline	centreline	periphery
0.6/average true velocity	2.0	1.94	3.53	0.97	0.94
30/TLPG	1.52		2.65	0.73	

We observe the two estimates for the factor of safety, independently calculated, are similar for normal daytime (1.9 versus 1.5), normal nocturnal (3.5 versus 2.7) and NTG daytime eyes (0.9 versus 0.7). Importantly the fact that these two independent safety factors, which are based on very different criteria (one safety factor is based on macroscale pressure gradient, while the other is based on microscale knowledge of dynein motor function), agree reasonably well, suggest that either identifying the axon fluid velocity leading to stalling of dynein motors, or identifying the macroscale pressure gradient along a peripheral nerve that leads to interruption of axonal transport, are essentially equivalent functional criteria. This helps us provide a firmer basis to our understanding of ‘axonal’ damage leading to disruption of axonal transport in the region of the laminar cribrosa. It also helps to build confidence in the model for predicting fluid transport within the normal and abnormal human eyes.

### Using the model to explain IOP elevation with a tamponade

The treatment of choice for (rhegmatogenous) retinal detachment is usually vitrectomy followed by insertion of either a gas or silicone oil tamponade. The purpose of the tamponade is to hold the retina against the retinal pigment epithelium so that it may reattach. Silicone oil is usually employed when a long-term tamponade is required, as gas tamponades tend to be resorbed relatively quickly (i.e. sulfur hexafluoride (SF_6_) resorbs in less than 14 days, and perfluorocarbon octafluoro-propane (C_3_F_8_) in less than 65 days [90]), which is often an insufficient time to promote complete retinal reattachment. Here we only consider silicone oil tamponades.

Silicone oil tamponades are not without their drawbacks. It is has been found that in up to 25% of cases (e.g. 9 of 36 enucleated eyes examined by [91], and 14 of 74 enucleated eyes examined by [92]) the silicone oil made its way from the vitreous chamber, across the optic disc into optic nerve head, and in some cases extends down the optic nerve. In at least a few reported cases, silicone oil has managed to find its way along the optic nerve and into the ventricles e.g. [93]. The extent of the migration appears to increase with time [92], and with elevated IOP [91, 94]. Silicone oil has also been found in the retina, choroid, ciliary body and the anterior outflow pathways [91, 95]. This data demonstrates that silicone oil eventually penetrates every outflow pathway along which aqueous fluid normally escapes the eye. And importantly, we mention that this observation also provides evidence that aqueous fluid is transported from the eye and along the optic nerve—for where silicone oil travels, so too does aqueous.

Initially it is somewhat surprising that silicone oil migrates from the vitreous chamber, as oil and water generally do not mix. Even though silicone oil has a low surface tension [96], it is hydrophobic, so in an aqueous tissue environment the silicone oil-water interface would be expected to have a large surface tension due to the water. The surface tension of water at the interface would prevent the silicone oil from migrating through small pores in the extracellular matrix of the tissue. This is normally demonstrated by calculating (by means of the Laplace-Young equation [97]) the pressure gradient required to move an oil droplet along a water-filled capillary of variable diameter. Indeed the expectation that there is no silicone oil migration across the optic disc is confirmed by experimentally pressure testing static, but otherwise normal, enucleated eyes filled with silicone oil at 40 mmHg, for up to 16 weeks [98].

However a very different outcome is obtained *in vivo*, as migration of silicone oil does occur in a matter of several months. It seems most likely that for *in vivo* migration to occur, surface tension at the oil water interface decreases over time (from the high surface tension of water at the silicone-oil interface towards the very low surface tension of the silicone oil [96]), due to the partitioning of ‘surfactant molecules’ to the oil-water interface. The surfactant molecules at the oil-water interface may arise from natural biologic molecules in the aqueous (examples of natural biologic surfactants are fibrinogen, fibrin, gamma globulins, acidic alpha-1-glycoprotein and very low density lipoprotein [99]). The surfactant molecules may also arise from additives to the silicone oil itself, from detergents on the instruments used in the surgical operation, or from solvents used by the surgeon during removal of the silicone oil from the eye. It is reported that emulsification of silicone oil *in vivo* typically takes 5 to 24 months with a mean of 13.2 months [100]. Emulsification also requires some continuous kinetic energy input (which *in vivo* is provided by eye movements), but once emulsification starts, the droplets then become progressively smaller and smaller, with ongoing kinetic energy input [99].

So the rate of silicone oil migration through tissues is controlled by the rate of kinetic energy input and the rate of accumulation of surfactants at the oil-water interface (note: more extracellular surfactant proteins are available to lower the interfacial surface tension in proliferative and inflammatory states, so proliferative and inflammatory states would be expected to speed emulsification). The rate of silicone oil migration is also driven by the magnitude of the pressure gradient within the interstitial pore spaces in the extracellular matrix containing the water and oil droplets. So one would expect that silicone oil migration is more likely in eye tissues that have an elevated pressure gradient. As we know, an important component of elevated pressure gradients in eye tissues in elevated IOP.

Most interestingly, one of the most important clinical issues regarding use of silicone oil tamponades is the post-operative management of elevated IOP [101–104]. The reason for the elevated IOP is largely unknown. But clearly introduction of the silicone oil tamponade itself appears to increase IOP, while removal of the silicone oil tamponade decreases IOP again [105]. It is therefore of considerable practical interest to understand why a tamponade increases IOP.

We ask: can our fluid transport model for the normal eye provide an explanation for elevated IOP with silicone oil tamponade based on our model for physiology of fluid outflow from the eye? Given that our flow model has about half of the aqueous production exiting through the retinal pigment epithelium, it seems reasonable to suppose that a silicone tamponade could interfere with aqueous outflow across the retinal pigment epithelium, and this may cause an elevated IOP. It is known that silicone oil lies very close to the retina, with the measured ‘sub-retinal fluid thickness’ being less than 5 to 10 microns [106]. This close apposition of silicone against the retinal tissue provides little opportunity for fluid to be transported to the site of fluid outflow where the silicone oil is in close apposition to the retina. Given this situation, here we effectively presume the silicone oil *prevents* fluid outflow across the underlying retinal pigment epithelium, wherever the retina is ‘covered’ by silicone oil.

Given this proposal, if we can first define the fraction of retinal surface area covered by the silicone oil, then we can easily employ our model to estimate the change in outflow facility through the retinal pigment epithelium, and then predict the change in eye IOP. In the following, we adopt this modeling strategy to test the idea that silicone oil prevents outflow through the retinal pigment epithelium beneath the retina when the retina is in close apposition with the silicone oil tamponade.

Fortunately, the shape and surface coverage of silicone oil tamponade in the normal human eye at different ‘filling fractions’ have been defined by Isakova et al. [107]. Employing the diagram shown in Fig 7 of [107], we estimate that fractional retinal surface area reductions of 20%, 30%, 45% and 68% occur for silicone oil vitreous volume filling fractions of 45%, 60%, 75% and 90% respectively. Now assuming reductions in outflow facility across the retinal pigment epithelium in direct proportion to the reduction in surface area due to the silicone oil, and assuming aqueous production is unchanged, we employ the model to predict the IOP after introduction of the silicone oil tamponade. The findings for these analyses are shown in Table 7.

**Table 7:** Predicted changes in IOP for different silicone oil volume fractions, assuming unchanged aqueous production using the flow model.

IOP predicted by model BC2 following vitrectomy and silicone oil tamponade in vitreous cavity (aqueous production unchanged)				
Volume fraction of silicone oil in vitreous cavity	45%	60%	75%	90%
Reduction in retinal surface for outflow	20%	30%	45%	68%
Predicted IOP elevation (mmHg) above 15 mmHg	2.0	3.4	4.3	15.0
Final predicted IOP mmHg (initial IOP 15 mmHg)	17.0	18.4	21.3	30.0

More specifically, we can use the model to examine the data presented by [105]. Jonas et al. provides a detailed report on the effect of introducing a silicone oil tamponade on the IOP of 198 patients (mean age 51.9 ± 18.3 years). All operations were completed by two surgeons, using the same technique on similar numbers of patients, so operator variability is reduced [105]. The initial IOP for the group was 12.9 ± 4.4 mmHg (median 13 mmHg). After introduction of the silicone oil tamponade the IOP rose to 16.1 ± 5.5 mmHg (median 16 mmHg). Each patient had four eye examinations, the first examination 2 weeks post-operation. Measured IOP did not change significantly between the first, second, third and fourth follow-up visits. Importantly, after removal of the silicone oil, the IOP dropped significantly by 3 mmHg to 13.3 ± 3.8 mmHg (median 13 mmHg), as reported at the first examination after removal of the silicone oil (i.e. within seven weeks of removal). The IOP remained statistically the same during the next follow-up examination (13.9 ± 5.0 mmHg) [105].

Importantly, it was also clear that the decrease in IOP upon removal of the silicone oil is significantly correlated to the increase in IOP upon the introduction of the silicone oil—‘*the higher the IOP before silicone oil removal*, *the more marked was the decrease in IOP after silicone oil release*’ [105]. The Pearson correlation coefficient was found to be 0.79 [105], which is very high given the complicating factors of age and the variety of disease states warranting the surgical intervention. Of the 177 patients examined at the first visit post-silicone oil removal, only 6 (3.4%) had an IOP greater than 21 mmHg (3 of these patients had been previously diagnosed with glaucoma). In patients with persistently elevated IOP, all could be reduced to the normal pressure range using topical anti-glaucoma medication [105].

We now attempt to model the data reported in [105]. We start with the model for the normal eye in the upright position and first reduce the aqueous production rate by 11.5% to achieve an initial IOP of 13 mmHg. We then reduce $C_{rpe}^{SL}$ until the model predicts a 3 mmHg elevation in IOP and find $C_{rpe}^{SL}$ equals 0.33 (i.e. $C_{rpe}^{SL}$ is reduced from the original value of 0.5 to 0.33). In other words, it appears as though the median amount of silicone oil introduced into the eyes effectively reduced the retinal surface area by 34% (1–2*0.33). Now from Fig 7 in [107], we predict the average silicone oil vitreal filling fraction is close to 65%. Jonas et al. also reports that 10.1% of patients had an IOP greater than 21 mmHg, 4.9% had an IOP higher than 30 mmHg and 1.5% had an IOP higher than 40% [105]. We find that if $C_{rpe}^{SL}$ equals 0.19, then the predicted IOP is 21 mmHg. Again Fig 7 in [107] allows one to predict for an IOP greater than 21 mmHg the average silicone oil vitreal filling fraction is greater than 85%. Similar calculations can be done for 30 mmHg and 40 mmHg. The results are summarized in Table 8.

**Table 8:** Analysis of data presented in Jonas et al. (2001), predicting volume filling fraction from IOP and retinal surface coverage.

Aqueous production rate adjusted model, all other parameters unchanged				
IOP with tamponade (> X mmHg)	>16	>21	>30	>40
Fraction of patients population (‘third visit’)	50%	13.9%	5.8%	2.3%
Predicted retinal surface area coverage by silicone oil	>34%	>62%	>83%	>92%
Predicted volume filling fraction by silicone oil	>65%	>85%	>96%	>98%

On the basis of the analysis presented here using the model, it is apparent that over-filling the eye with silicone oil is predicted to lead to significant increases in IOP, as observed clinically.

### Using the model to explain IOP elevation in Schwartz-Matsuo syndrome

In the previous section, we employed the model to investigate the effect of obstruction of outflow across the retinal pigment epithelium by silicone oil tamponade. Here we employ the model to investigate the effect of obstruction of outflow across the anterior pathways observed in Schwartz-Matsuo syndrome [108]. As explained by Matsuo, Schwartz-Matsuo syndrome is characterized by anterior rhegmatogenous retinal detachment with secondary occurrence of elevated IOP, with ‘aqueous cells’ in the anterior chamber. The aqueous cells are most likely photoreceptor outer segments that have separated from the detached retina, which have then found their way into the anterior chamber. Once in the anterior chamber the separated photoreceptor outer segments proceed to clog the anterior outflow pathways, explaining the elevated IOP.

This syndrome provides an opportunity to test the model predictions of IOP elevation given different reductions in the measured outflow facility through the trabecular meshwork for patients with this syndrome. To do this, we take the IOP and outflow facility measured for the 23 Schwartz-Matsuo patients that have a complete IOP-outflow facility measurement details for both pre-operation and post-operation, reported in Table 2 of [108].

We find the pre-operatively the mean IOP is 38.3 mmHg ± sd 11.3 mmHg, while the mean measured outflow facility was 0.077 ± sd 0.047 microlitres/min/mmHg. Post-operatively, the mean IOP is 14.9 mmHg ± sd 2.57 mmHg, while the mean measured outflow facility was 0.260 ± sd 0.067 microlitres/min/mmHg. We first tested the model simply by taking the same ratio of mean measured outflow facilities pre and post operation, and applying that same ratio to be the ratio of pre and post operation $C_{ap}^{SL}$ in the model. Specifically, we reduce $C_{ap}^{SL}$ from 0.5 to 0.148 (which approximately equals (0.077/0.260) × 0.5). All other parameters in the model remain unchanged.

Applying this modification to the model predicts a mean pre-operative IOP of 32.9 mmHg, while the measured mean IOP of 38.3 mmHg ± SEM 2.36 mmHg. Of course, the model predicts a normal IOP of 15 mmHg post-operatively, while the measured post-operative IOP is 14.9 mmHg ± 2.57 mmHg. It is clear that the predicted mean IOP pre-operation is a little over ± 2.0 standard errors of the mean for the measured mean IOP, while the post-operative mean IOP is within ± 2 standard errors of the mean for the measured mean IOP. However, we do observe that if the mean aqueous production rate is slightly higher (by about 4.5%) so the initial IOP becomes 16 mmHg due to increased aqueous flow (16 mmHg (within ± 2 SEM on the estimated mean IOP of 14.9 mmHg), then the predicted pre-operative IOP is 39.0 mmHg, which is close to the measured mean pre-operative IOP elevation.

In an attempt to again analyse this problem, we try a third approach. If we reduce $C_{ap}^{SL}$ from 0.5 to 0.12, the model predicted mean IOP is 38.3 mmHg. But we see this value of $C_{ap}^{SL}$ equal to 0.12 is entirely consistent with the experimental data reported in Table 2 of [108] based on an analysis of the uncertainty propagation (assuming pre and post outflow facilities are uncorrelated). For the SEM of the ratio of mean outflow facilities pre and post operation is ± 0.041. This means this value of $C_{ap}^{SL}$(i.e. 0.12), which delivers exactly the same pre-operative IOP elevation for both measured and predicted IOP, is within ± 1 SEM of the mean estimate of the ratio of outflow facilities pre and post operation (i.e. 0.148–0.12 = 0.028 is less than one SEM (0.041) from the mean).

Either of these two later approaches bring the model predicted IOP into agreement with the observed IOP within 1 or 2 SEMs, so on this basis we deem the model to be capable of providing a good fit to the Matsuo data [108]. This is especially so considering the variation in location and size of rhegmatogenous detachment, the differences in the numbers of aqueous cells observed for each patient, the considerable uncertainties associated with tonographic measurement of outflow facility, and the normal biological variation between individuals.

## Discussion

This paper describe the development and calibration of an axisymmetric, three dimensional fluid outflow model for the human eye. The fluid outflow in the model is pressure dependent, being regulated according to the model described in detail in [34]. The geometric anatomic details for the model human eye (S1 Table), as well as parameters employed for the model human eye (see Table 1), have been inferred from data reported in the literature.

While geometry data is based on dimensions for a normal human eye, the parameter estimates necessarily include animal data. For the vitreous humor hydraulic conductivity, we rely on high quality bovine data. For the region of the optic nerve head, there is a heavy reliance on the high quality data collected for the dog as reported by Morgan and colleagues. Additional data is inferred from measured hydraulic conductivities of white and gray matter in the brains of the cat and rat. The use of animal data for parameter estimation represents one important limitation of the model for humans proposed here.

Perhaps as importantly, some of the parameters have not been directly measured in the human or animal eyes. These parameters have been inferred. For example, this is the case for the important hydraulic conductivity estimates for the laminar tissues within the optic nerve head (i.e. the prelaminar tissue, laminar cribrosa tissue and the immediate (region of partially myelinated) retrolaminar tissue. However, the large potential uncertainty in this parameter estimate has been greatly reduced by anchoring the hydraulic conductivity for the myelinated optic nerve to be that measured for white matter in the brain of the cat, and then fitting relative hydraulic conductivities in the optic nerve head to match the fluid pressure profile along the optic nerve head into the optic nerve as reported in [30] for the dog.

Having completed the model calibration for a normal IOP of 15 mmHg in the anterior chamber of the eye, we find there is a small but significant pressure drop across the retina (estimated to be about 1.1 mmHg), which helps to press the retina against the retinal pigment epithelium. Our model predicts a pressure drop across the vitreous towards the optic nerve, however the predicted magnitude of the pressure change is smaller than is measurable by current techniques. Our model predicts a pressure drop of about 0.15 mmHg from the anterior chamber to the posterior of the vitreous humor in the human eye, representing about a 1% decrease in fluid pressure close to the optic disc relative to IOP measured in the anterior chamber. But there is uncertainty in the vitreous hydraulic conductivity measured in bovine eyes by Xu et al. being applied to human eyes, and clearly the model can be updated when new high quality experimental data for human eyes become available.

### Model predictions for silicone oil tamponade and Schwartz-Matsuo syndrome

How the introduction of a silicone oil tamponade causes elevated IOP is largely unexplained. For example, Honavar et al. could attribute the observed elevation of IOP to factors associated with the complicated retinal detachment in only 30% of cases, while for the remaining 70% of cases the elevated IOP was ‘*attributed directly to silicone oil*’ [102]. Jonas et al. could only explain about 10% of cases of elevated IOP, while for the remaining 90% ‘*no obvious reason for increased IOP was detected*’ [105]. How can we explain the remaining cases of IOP elevation?

In this context, we observe that our 3D outflow model for the normal human eye has about half of the outflow passing through the retinal pigment epithelium, and with the introduction of a silicone oil tamponade that lies in very close apposition to the retina, our model lends itself to testing the hypothesis that the silicone oil tamponade in very close proximity to the retina interrupts fluid outflow across the retinal pigment epithelium, thereby offering a possible explanation for the previously unexplained elevation of IOP.

We tested the model using the data reported in [105], as the Jonas et al.’s data offered the advantages that is was collected by only two surgeons, and so had less inter-operator variability than many studies. Further, there appears to be sparing use of medications to lower post-operative IOP (the use of IOP lowering medication now appears to be a commonplace in many studies of this type e.g. [102, 103]). And finally, Jonas et al. presents detailed reporting of pressure elevation for the group and subgroups of patients. For example, they include the median pressure rise for the whole group upon introduction of the silicone oil, and the median pressure decrease for the whole group upon its removal (reporting median elevations in IOP is important, for mean pressure elevations may be dominated by a few higher IOPs).

Jonas et al.’s data shows that the median IOP became elevated upon introduction of the silicone oil, and that the median IOP decreased back to the pre-existing IOP upon removal of the eye. And importantly for establishing a causal interaction, Jonas et al. found that for individual eyes, the magnitude of the increase in IOP is strongly correlated with the magnitude of the decrease in IOP upon removal of the silicone oil.

We also mention that 8 of the patients (4%) in the group had a history of ‘glaucoma’ (5 had been diagnosed as having glaucoma, and 3 with elevated IOP for unknown reasons), and were taking topical anti-glaucoma medication prior to the vitrectomy surgery [105]. Following silicone oil removal, glaucomatous optic nerve damage was detected in 14 eyes (7.1%) (i.e. and an additional 9 eyes (4.5%) developed glaucoma), which serves to highlight the risk of permanent eye damage associated with elevated IOP while using silicone oil tamponade.

After decreasing aqueous production 11.5% to obtain the pre-operative IOP of 13 mmHg reported by Jonas et al., the model appears to be successful in predicting the rise in IOP with fractional filling of the vitreous chamber (see Table 8). Though the fraction of silicone oil filling the vitreous chamber was not measured by Jonas et al. (to do this accurately would require MRI imaging of patients, which is not clinically indicated for treatment of retinal detachment), it appears as though the model predicted fractional filling volumes that are reasonable based on reported pressure elevations. They are, for example, in line with expected levels of silicone oil filling (e.g. 60%+) reported in [107].

The clinical implication of the model predictions is that one should not overfill the eye with silicone oil, where over-filling is taken to mean using more silicone oil than is necessary to achieve the intended tamponade effect on the detached retina. For example, 65% filling of the eye with silicone oil results in a predicted modest 3 mmHg rise in IOP (to 16 mmHg), which minimizes any potentially deleterious side-effects associated with elevated IOP. On the other hand, 98% filling of the vitreous chamber with silicone oil results in a predicted 27 mmHg rise in IOP (to 40 mmHg), which clearly should be avoided if at all possible, and immediately treated with medication if necessary. Sometimes elevated IOP is not well controlled by medication, and when high fractional filling of the eye with silicone oil appears necessary and this results in a persistent elevation of IOP, surgeons may sometimes resort to removal of at least some of the silicone oil from the eye [101].

In their review of silicone oil tamponade and IOP elevation over four decades, Ichhpujani et al. identify four explanations for IOP elevation: “*(i) pupillary block by silicone oil excites angle-closure glaucoma*, *(ii) emulsified silicone oil may sweep into the trabecular meshwork causing secondary open-angle glaucoma*, *(iii) inflammation or exacerbation of pre-existing glaucoma and (iv) overfill causing filling of the anterior chamber leading to open-angle glaucoma by mechanical obstruction of outflow”* [109]. This clearly recognises overfilling of the eye as undesirable because it may cause pupillary block or it may result in the silicone oil entering the anterior chamber, where silicone oil droplets causing mechanical obstruction of outflow through the anterior pathway. But these four causes do not explain the fact nearly all eyes with a silicone tamponade experience some chronic IOP elevation, even if it be small (e.g. Jonas et al. reported a 3 mmHg rise in the median IOP for the group, and a 3.2 mmHg rise in the mean IOP for the group). In contrast, our model does explain this generalised IOP elevation in the treatment population. Even if this general IOP elevation is deemed clinically ‘insignificant’ from the viewpoint of clinical side-effects of silicone oil tamponade, IOP elevation is of considerable physiological significance for understanding the physiological basis for the elevated IOP. Further the model does predict clinically significant IOP elevations given sufficient filling fraction of the vitreous chamber with silicone oil, even in the absence of the four explanations for IOP elevation described by Ichhpujami et al.

While maintaining normal outflow through the anterior pathways (i.e. through the uveoscleral and trabecular meshwork pathways), we have successfully explained IOP elevation using silicone oil tamponade by the reduction in outflow across the retinal pigment epithelium. We then explored if it was possible to use the model to explain the IOP elevation when outflow through the anterior pathways is reduced, while maintaining normal outflow across the retinal pigment epithelium. Though Schwartz-Matsuo syndrome is relatively rare, measurements on these eyes made before and following treatment of this syndrome provides us with an opportunity to again test the predictive capability of the model.

Schwartz-Matsuo syndrome is characterized by anterior rhegmatogenous retinal detachment (detachment is generally close to the ‘ora serrata’) with secondary occurrence of elevated IOP. Schwartz-Matsuo syndrome normal arises as a sequela to blunt traumatic injury of the eye. It is believed that photoreceptor outer segments separate from the detached retina, and make their way into the anterior chamber, where they clog and so reduce outflow through anterior pathways, explaining the observed elevation of IOP [108]. The elevated IOP normally spontaneously resolves upon successful treatment of the retinal detachment [108].

We find that a value of $C_{ap}^{SL}$ equals 0.12 in the model explains accurately the same pre-operative IOP that that has been measured and predicted by the model. This value for $C_{ap}^{SL}$ is within ± 1 SEM of the mean estimate for $C_{ap}^{SL}$ (i.e. 0.148). We deem this to good model fit, and conclude that the 3D flow model can successfully explain the observed IOP elevation in Schwartz-Matsuo syndrome.

In other words, the model is capable of explaining both the IOP elevation when the outflow pathway across the retinal pigment epithelium is partially blocked, and the IOP elevation when the anterior outflow pathways are partially blocked. As far as the authors are aware, no previous 3D outflow model for the human eye has successfully explained pressure elevation with a silicone oil tamponade and for Schwartz-Matsuo syndrome, or are capable of doing so. Most importantly for the purposes of employing the model in a predictive role, the success of the model as described above helps to build further general confidence in the proposed physiological model of pressure-dependent outflow from the eye (as embodied in the structure of the outflow model), and further, in the model parameters chosen to represent the normal human eye (as described above for the model’s parameters). We employ the model to analyse the TLPG in the eye.

### Model predictions for translaminar pressure gradient

Unfortunately many critically important operating states within the body are not directly accessible to clinical measurement. For examples, peripheral blood resistance or the degree of sympathetic outflow activation, to choose two state parameters of the many dozens of such parameters for the cardiovascular system alone. So it is for the eye system, with the TLPG being a state parameter for the eye that is not directly measureable clinically. But in many cases, carefully calibrated computational models of the physiological system can help infer the system state, and so provide estimates of variables that are not directly accessible to measurement. This is one of the principal clinical advantages of developing computational models. A major goal of this paper was to show it is possible to more accurately estimate the translaminar pressure gradient using a computational model of outflow by employing a three dimensional model of the eye that includes fluid flow across the optic nerve head.

Even though the translaminar pressure gradient is known to the major risk factor for glaucoma for some time, it presents a difficult problem to clinically measure the translaminar pressure gradient. Most often resort is made to measuring the IOP and the lumbar CSF pressure, and crudely estimating the translaminar pressure gradient by subtracting these two measurements [32, 67, 110, 111]. Though IOP is normally measured in the upright (sitting) position, a lumbar puncture pressure measurement in normally made in the lateral decubitus position (note that lumber puncture pressure is approximately the same as the cranial CSF pressure in the lateral decubitus position [67]). But both the IOP and cranial CSF pressure are posture dependent. Cranial CSF pressure decreases to negative pressures in the upright position relative to CSF pressure in the lateral decubitus position, which more careful studies take into account when estimating intracranial fluid pressure (e.g. [67]). Attempts are then made to correlate these gradient estimates with the prevalence of types of glaucoma in the population [32, 110, 111].

Direct measurement of the translaminar pressure gradient can only be done in animals, and it requires extraordinary levels of technical skill to do this accurately. Fortunately high quality data is available for the dog [30, 37]. We use this data to calibrate our computational model for the human eye, and find that we can closely replicate the reported normalized pressure profiles from the optic disc along the optic nerve. In the model, we included a 3 mmHg ‘floor pressure’ on the CSF pressure in the subarachnoid space around the optic nerve, and we calibrate the hydraulic conductivity of the pial membrane and nearby tissue to reflect the observed increased resistance to outflow across the pial membrane and nearby tissue, as reported by [37].

The model predicts the translaminar (cribrosa) pressure gradient for the normal human eye is 19.7 mmHg/mm. This compares with a ‘baseline’ translaminar (cribrosa) pressure gradient for the human eye predicted by Ayyalasomayajula et al. (i.e. estimated at baseline model parameters) of 39 mmHg/mm (which assumed the pre-laminar tissue had “*the same baseline properties as the retina-Bruch’-choroid complex*” [112]), while the measured translaminar (cribrosa) pressure gradient for the dog eye of 23 mmHg/mm [37].

We also mention that by looking to the dog data (e.g. [37]), Balarainasingam et al. tried to very approximately estimate the translaminar pressure gradient for the human eye [113]. Balarainasingam et al. predicted that for humans the mean pressure gradient across the whole thickness of prelaminar and laminar cribrosa tissues to be about 20 mmHg/mm, while for the same pressure gradient across the laminar cribrosa alone, the translaminar (cribrosa) pressure gradient was estimated to be 33 mmHg/mm [113]. Of course, neither assumption is correct.

The TLPG predicted by our model (after fitting the same shape of normalised pressure gradient observed in the dog to our human model by adjusting relative permeabilities of various tissues in the optic nerve head, we find the 20 mmHg/mm), is in reasonably close agreement with the TLPG observed in the dog (23 mmHg/mm).

Upon assuming the supine position, our model predicts the normal TLPG decreases substantially to 14.7 mmHg/mm (i.e. by about 25%). It is also of considerable interest to estimate what happens during sleep. We re-calibrated the model to the data reported by Nau et al. (for older adults, mean age 59 years) and demonstrated a good fit to their data. We then calculated the predicted nocturnal TLPG for the IOP measured in the sitting position (as done by Nau et al.) and in the supine position (which is presumably more closely represents conditions during sleep). Based on the model modified to fit the Nau et al. data, we predict the upright nocturnal TLPG to be 13.4 mmHg/mm and the supine nocturnal TLPG to be 11.3 mmHg/mm, the latter about 40% less than the normal adult upright daytime TLPG.

We defined a ‘factor of safety’ for acute interruption of axonal transport in the human eye. A factor of safety is simply a number that helps one better appreciate the probability of some adverse event. For example, we note that Biewener defined a factor of safety against bone fracture as the ultimate stress a bone is capable of withstanding, divided by the ‘working stress’ for that bone (where the working stress is the maximum stress experienced in the long bones while an animal is running, hopping, jumping or galloping, as the case may be). This definition is the standard definition for a (single) structural factor of safety.

Interestingly, Biewener found that the factor of safety against bone fracture was about 2.0 to 4.0 [72], and he found this factor of safety was fairly constant, being independent of animal size e.g. a hopping rat, a jumping dog, a jumping horse and charging elephant, all have a factor of safety against fracture of about 2.0 (see Fig 1 in [72]). Remarkably, the most common magnitudes chosen by modern design codes that employ a single ‘factor of safety’ to be used for engineering design, usually range between 2.0 and 4.0. To provide more context on factors of safety, we mention that most current engineering design codes use multiple ‘partial safety factors’, or advanced codes use ‘reliability based design’ methods [114], which employ probability distributions to represent generalized loads and represent generalized resistances, primarily because a single value for the factor of safety may not allow for a sufficient level of risk assurance, which may be required in more risk critical situations. But for our application, using a single factor of safety is a reasonable first step.

For our particular application, we first define a ‘factor of safety’ as the pressure gradient of 30 mmHg/mm (i.e. the threshold pressure gradient after 17 hours of exposure reported by [71] for interruption of axonal transport in the vagus nerve) divided by the actual (or model predicted) translaminar pressure gradient, which has a similar generic form to the factor of safety against bone fracture described above. When this factor of safety is equal to one or less than one, axonal transport is acutely compromised, and when the factor of safety is significantly greater than one axonal transport is not compromised.

Interestingly, we see that for the normal eye in the upright posture, the estimated factors of safety is about 1.5 to 2.0 rising to somewhat over 2.7 in the supine position at night (see Tables 5 and 6). However for the model NTG eye, the factor of safety is always less than 1.0 (0.65 to 0.97), suggesting the development of a glaucomatous neuropathy is likely, and becoming more likely with advancing years (see details Tables 5 and 6). Importantly, we observe this range for factors of safety against acute interruption of axonal transport in the human eye is similar to the factors of safety reported by Biewener for bone, and they are also similar to those used by engineers in engineering design practice. This provides some level of reassurance that the estimated factors of safety by the proposed definition against acute interruption of axonal transport are plausible.

### Using the model to predict disruption of axonal transport

Axons contain axoplasm that has a measurable Newtonian viscosity. Band et al. first proposed that the fluid in the axoplasm flows along axons under the influence of a pressure gradient [29]. Using a micro-viscosity measured for the axoplasm found in the sciatic nerve of cats [115], they applied Poiseuille’s Law for capillary flow to each axon (assuming the axon membrane is like a capillary), and allowing for fluid leakage into, between and from axons, Band et al. calculated a rate of flow from the eye to the cerebrospinal fluid in the subarachnoid space around the optic nerve. While we believe many of the basic concepts behind the analysis are correct, unfortunately the predictions of the Band et al. analysis conflict with experiment observations. For example, Band et al.’s analysis does not reproduce the experimentally observed TLPG [30, 37]. Rather Band et al.’s predicts constant pressure through the ONH and into the optic nerve equal to the IOP, apart from a very narrow strip of tissue 50 to 150 microns wide next to the surface of the optic nerve, where axoplasmic fluid flow occurs (see Figs 2 and 3 in [29]). What we believe Band et al. do have correct is that water enters the axons within the retina (presumably via permeation of water through the cell membrane, but also including permeation through aquaporins in the cell membrane [116]), which provides the source of fluid moving through the axonal cytoskeleton. Water leaves the axons once the axons have passed through the laminar cribrosa (again through via the cell membrane and aquaporins), across the pial membrane to join the CSF in the subarachnoid space surrounding the optic nerve.

We believe the flow of saline water and advected solutes through the axonal cytoskeleton is better represented using a ‘plug flow’ model (i.e. a Darcy flow) rather than a paraboloid flow velocity profile (i.e. a Poiseuille flow), because the axoplasm is filled with a regular spacing of microtubules and neurofilaments (which have side-chains: see for example Fig 4 in [117]). These filaments within the axon offer considerable resistance to fluid flow along the axon, and this prevents a paraboloid velocity profile developing across the axon. Nevertheless, there is a plug-type flow of saline water and advected solutes through the filamentous network that is the axonal cytoskeleton within the retinal ganglion cell axons. It is this plug flow of fluid that is the average true fluid velocity inside the axon, and it this quantity that is employed in Eq (7).

There is a ‘structural problem’ to be addressed arising from this fluid flow through the axonal cytoskeleton, for the drag force caused by the fluid flow past the microtubules and neurofilaments making up the axonal cytoskeleton (together with any molecules and cell structures attached to the cytoskeleton) creates a ‘body force’ within the axoplasm. This body force within the axons creates (mainly) a shear force within the filament network that needs to be transferred sideways to the cell membrane. To facilitate the develop of shear stress, the shear stiffness of the neurofilament network may be increased by phosphorylation of the neurofilaments, as phosphorylation increases the osmotic pressure between the neurofilaments [117]. Phosphorylation also promotes divalent cross-bridging between filaments, which presumably also enables greater transfer of shear stress to the cell membrane [118]. We note in passing that phosphorylation of neurofilaments is particularly prominent where hydraulic conductivities are lowest, and fluid flow induced body forces on the filament network are largest (i.e. in the optic nerve head) [39].

We know cell membranes are delicate structures, so once the shear load is transferred from the cytoskeleton to the cell membrane, there is a danger of the cell membrane being torn apart by the tensile force transferred to it. So on this basis, we might expect the cell membranes of the axons to be structurally supported in some way. And indeed, we find that astrocytes extend processes, packed with intermediate filaments (which are strong in tension), which insinuate themselves around and between the axons and pick up the load transferred to the cell membrane. These astrocyte processes provides the structural support required to carry the load induced by the fluid flow through the axons. This anatomic detail is beautifully illustrated by the histological images of the astrocytes within the laminar cribrosa shown in Figs 5, 6 and 7 in [89], which is revealed in greater detail by the transmission electron micrographs shown in Figs 11, 12 and 13 of [89].

Having transferred the load to the filament network within the astrocyte processes, how are the astrocyte processes supported? The load is then transferred along the astrocyte processes to layers of astrocyte cell bodies, which line the canals in the laminar cribrosa (see the beautiful drawing depicting the arrangement of astrocytes in the optic nerve head: particularly relevant is Schema 5 shown in [119] for the lamina cribrosa). From the astrocyte cell bodies, the load is transferred to the collagen in the canal walls, and then to the collagen ‘beams’ that make up the laminar cribrosa. The lamina cribrosa beams then connect to the adjoining sclera. In this way, the body (or fluid drag) force loading arising from the fluid flow within the axons, is transferred from the neurofilament network to the sclera. What is of most interest here is the fact if we assume there is an axoplasmic flow generating a drag force on the neurofilaments, then the detailed anatomy of the laminar cribrosa is logically explained i.e. the structural details of the optic nerve head fit this presumed functional need. This also helps to build confidence that there is axoplasmic flow of fluid through the axonal cytoskeleton, along the retinal ganglion cell axons.

In this context we mention that the mouse has no laminar cribrosa, but rather the axons are supported by a ‘glial laminar’ (see for example Fig 4 in [120]). Interestingly we observe the diameter of the mouse optic nerve at the glial laminar is about the same size as the canals in the human laminar cribrosa (which are 40 microns to 65 microns across [89]). Presumably astrocytes can structurally support the fluid forces generated within a bundle of axons up to about 65 microns in diameter, but they cannot do so for larger diameter bundles of axons. An interesting corollary of this observation is that if the TLPG were to become chronically elevated, we would then expect that the laminar cribrosa would need to remodel to meet the structural need for astrocyte support of axon bundles. In fact, there is evidence for remodeling of the laminar cribrosa when the IOP is chronically elevated, including a 44% to 82% increase in connective tissue volume, a 17% to 48% increase in laminar beam number in the laminar cribrosa, and an increased laminar cribrosa thickness [121].

There are a number of modeling limitations in our three-dimensional fluid outflow analysis for the eye. First there are short-comings in our analysis of the TLPG, which is most relevant to calculation of factors of safety against axonal transport block. The hydraulic conductivity estimated for the optic nerve head is that for the whole tissue (i.e. axons and extra-axonal tissues i.e. including the extracellular matrix and all non-neuronal cells making up the laminar cribrosa). It could be that all the fluid moves along extra-axonal pathways (i.e. interstitial pathways through the extracellular matrix), or the reverse, all fluid moves through the axoplasm. In either case, the true fluid velocity would increase over that calculated above, as the cross-sectional area for fluid flow is reduced.

For the above analysis, we have effectively assumed extra-axonal hydraulic conductivity is the same as the axonal hydraulic conductivity, so the Darcy fluid velocity is the same in each compartment (note: this still allows true fluid velocity to be different for each compartment, depending on the estimated porosity of each compartment). While there is no evidence to discredit this assumption, and it is probably a reasonable assumption on functional grounds, there is no experimental evidence to support it either. Clearly additional experimental research is required to decide if there is a significant difference between interstitial hydraulic conductivity and axonal hydraulic conductivity.

It is also possible that an elevated rate of flow through the optic nerve head may ‘washout’ components of the filament network in axons by various mechanisms, so the permeability changes with time. There is some evidence to show that neurofilament composition in the optic nerve head does change with time following an abrupt elevation of IOP [17], but it not known if this results in changes in fluid permeability of the optic nerve head. We have not included any time dependent permeability changes in the model.

We mention again that the model developed here is for a rigid porous media, which by necessity means the initial geometry of the eye and initial strains within eye tissues does not continue to change further with changes in IOP, but remains fixed in space. Some more sophisticated models have fluid flow through the eye coupled with deformation of the solid phases [112, 121–126]. However, for IOP changes about normal IOP (15 mmHg), say in the range of 5 mmHg to 40 mmHg, generally average strains in the tissues optic nerve head are less than about 1% [112, 126, 127], (and possibly less than about 0.5% when starting from the normal IOP). Small strains of this magnitude are probably unlikely to significantly change the permeability of tissues in the optic nerve head as a result of strain. Though a different eye tissue, we note for example that corneal scleral permeability decreases by half when compressed by about 14% axial strain [128], while axial ONH strain is probably considerably less than 1%, suggesting the change in permeability taking into account strain in the extracellular matrix of the ONH is also likely to be small. But clearly a more accurate model would take changes in geometry with IOP and take the change in model parameters with strain into account.

A second concern relates to comparing factors of safety based on quite different experimental data. For example, the pressure gradients along the vagus nerve of the rabbit demonstrated interruption of anterograde axonal transport, while the dynein analysis in the optic nerve of mice focused on retrograde axonal transport. However, *in vivo* experiments on the macaque (*macaca fascicularis*) eyes demonstrate that elevated IOP interrupts both anterograde and retrograde axonal transport at the laminar cribrosa at about the same threshold [129]. And while all measurements relevant to the model are not provided in the paper by Minckler et al., it is apparent that the threshold IOP for axonal transport interruption of 25 mmHg (compared to a normal IOP of 10 mmHg to 14 mmHg), is broadly consistent with the factor of safety of about 2.0 on the TLPG, which provides some further reassurance about the appropriateness of the estimated factors of safety.

A third concern, when calculating a factor of safety for acute interruption of retrograde axonal transport, is the fact that experiments show there is a probability distribution for the velocity of retrograde transport [74, 85], rather than the single time-weighted value identified by [80] and successfully employed in a computational model developed by [76]. The existence of a probability distribution for retrograde velocities suggests that almost any net fluid flow along the axon at all will decrease retrograde transport, and the magnitude of this decrease in retrograde transport only grows continuously with increasing velocity of axonal fluid flow. Therefore the factor of safety for acute interruption of retrograde transport will decrease continuously as the velocity of axonal fluid flow increases, or increase continuously as the velocity of the axonal fluid flow decreases. This is both interesting and probably important for understanding the maintenance of normal axonal function. For clearly the TLPG varies over 24 hours, and so a fuller assessment of safety factors for chronic interruption of axonal transport would need to take into account the time variation of the velocity of axonal fluid flow. This would help answer questions such as: is less sleep correlated with greater likelihood of glaucomatous neuropathy, and are certain sports or exercise routines (e.g. involving head down positioning) correlated with greater likelihood of glaucomatous neuropathy?

Despite these shortcomings, it is nevertheless remarkable that the *macroscale estimate* for the factor of safety against acute interruption of the axonal transport based on macroscale TLPG, is similar to the *microscale estimate* for the factor of safety against acute interruption of the axonal neurofilament transport based on dynein motor mechanics. This agreement helps to build confidence in our 3D model for pressure dependent outflow from the human eye.

## Conclusions

In this paper we build a 3D PDE model for pressure dependent outflow from the human eye, starting from the previously published ODE model by [34]. We calibrate the model using the data available in the literature, which fortunately includes the data published by [30, 37] on the pressure distributions through the optic nerve head for the dog. This enables us to develop a reasonably accurate model of flow through the optic nerve head to the cerebrospinal fluid in the subarachnoid space surrounding the optic nerve. Assuming anterior outflow pathways are operating normally, we employ the model to explain why and by how much IOP becomes elevated upon the introduction of a silicone oil tamponade into the vitreous chamber. Then assuming outflow across the retinal pigment epithelium is normal, we employ the model to explain by how much IOP becomes elevated in Schwartz-Matsuo syndrome. This is the first time a pressure-dependent outflow model has successfully modelled both these conditions, which helps build confidence in its predictive capability. We calculate the TLPG in the upright and supine postures, and during day and night. Based on the data of [71] we define a macroscale factor of safety against acute interruption of axonal transport, and find that the estimated factor of safety varies with posture and the time of day. Based on the measurements by [80], which were been successfully incorporated by [76] in their model of axonal transport along the optic nerve of the mouse, the known mechanics of dynein motors, and our model estimated true fluid velocity along retinal ganglion cell axons, we compute another factor of safety for acute interruption of axonal transport of neurofilaments. For the normal eye, we find the two factors of safety, estimated by independent methods, both generate similar estimates for the factor of safety, being between about 1.5 and 2.0 during the daytime and 2.7 and 3.5 during the night-time. Interestingly, and perhaps reassuringly, the magnitude of the estimates for the factor or safety are similar to those estimated for bone fracture and employed in engineering design. For the case of normal tension glaucoma, the model estimates a factor of safety that are less than one, suggesting that glaucomatous disease progression is likely. While much remains to be discovered, we believe that the fluid outflow model for the human eye presented here may prove itself useful for many applications that are of both physiological interest when interpreting eye behaviour and clinical interest when managing eye disease.

## Supporting information

S1 Table Geometry for model of human eye. (DOCX)

S2 Table Hydraulic conductivities of various tissues. (DOCX)

S3 Table Change in IOP from ODE model as components of the eye are sequentially added to the 3D PDE flow model. (DOCX)
