# Supplementary material for: Estimating three-dimensional outflow and pressure gradients within the human eye
Source: PLoS One. 2019 Apr 9;14(4):e0214961. doi: 10.1371/journal.pone.0214961 (PMC6456205; doi:10.1371/journal.pone.0214961)
Supplement: S1 Table — (DOCX) [file pone.0214961.s001.docx]

**S1 Table. Geometry for model of human eye.**

|  | Eye Geometry | | |
| --- | --- | --- | --- |
| anatomic structure | model geometry | Experimental measurement | Reference |
|  |  |  |  |
| Internal diameter eye including retinal thickness | 23.6mm | emmetropic/normal eyes 23 mm ± sd = 0.4 mm long and 22.7 ± sd = 0.4 mm wide (excluding retina thickness) | [1] |
| Retinal thickness | 200 microns | ≈200 microns | [2] |
| Retinal surface area | 1,411 mm^2^ | 1,363 ± 160 mm^2^ | [3] |
| Eye volume | 6320 microlitres | 6500 microlitres for men, 6200 microlitres for women | [4] |
| Volume of anterior chamber | 150 microlitres | 170 microlitres ± 40 microlitres | [5] |
| Optic disc area/diameter | 2.0 mm^2^/1.6 mm | Corrected optic disc area ranges between 1.3 mm^2^ and 2.5 mm^2^ | Figure 3b in [6] |
| Optic nerve diameter | 3.2 mm | 3.2 mm | [7] |
| Optic nerve head length | 0.82 mm (superficial axon layer (0.195 mm) prelaminar region (0.245 mm) and laminar cribrosa (0.38 mm) and immediate retrolaminar tissue) | 0.6 to 1.0 mm | Figure 3 in [8] |
| Laminar cribrosa normal human eye | 0.38 mm | 0.391 mm ± 0.107 mm | [9] |
| Shortest distance from laminar cribrosa to the prelaminar tissue | 0.38 mm | 0.420 mm ± 0.110 mm  range 0.16 to 0.90 mm | [9] |
| Peripapillary scleral thickness normal human eye | NA (sclera not included in model) | 0.283 mm  ± 0.0.72 mm | [9] |
| Optic nerve diameter | 3.6 mm | measured 3 mm behind the globe, mean diameter 3.2 mm (95% range 2.5 to 4.0 mm) | [10] |
| Major axis length | 9.8 mm | in vivo eye, 9 mm to 10 mm | [11] |

**References**

1. Atchison DA, Jones CE, Schmid KL, Pritchard N, Pope JM, Strugnell WE, et al. Eye shape in emmetropia and myopia. Invest Ophth Vis Sci. 2004;45(10):3380-6. doi: 10.1167/iovs.04-0292. PubMed PMID: WOS:000224154800003.

2. Grover S, Murthy RK, Brar VS, Chalam KV. Comparison of Retinal Thickness in Normal Eyes Using Stratus and Spectralis Optical Coherence Tomography. Invest Ophth Vis Sci. 2010;51(5):2644-7. doi: 10.1167/iovs.09-4774. PubMed PMID: WOS:000277180500047.

3. Nagra M, Gilmartin B, Thai NJ, Logan NS. Determination of retinal surface area. J Anat. 2017;231(3):319-24. doi: 10.1111/joa.12641. PubMed PMID: WOS:000407654000001.

4. Silver DM, Geyer O. Pressure-volume relation for the living human eye. Curr Eye Res. 2000;20(2):115-20. doi: Doi 10.1076/0271-3683(200002)20:2;1-D;Ft115. PubMed PMID: WOS:000085484900007.

5. Labiris G, Gkika M, Katsanos A, Fanariotis M, Alvanos E, Kozobolis V. Anterior chamber volume measurements with Visante optical coherence tomography and Pentacam: repeatability and level of agreement. Clin Exp Ophthalmol. 2009;37(8):772-4. doi: 10.1111/j.1442-9071.2009.02132.x. PubMed PMID: WOS:000271311800004.

6. Moghimi S, Hosseini H, Riddle J, Lee GY, Bitrian E, Giaconi J, et al. Measurement of Optic Disc Size and Rim Area with Spectral-Domain OCT and Scanning Laser Ophthalmoscopy. Invest Ophth Vis Sci. 2012;53(8):4519-30. doi: 10.1167/iovs.11-8362. PubMed PMID: WOS:000307096400025.

7. Band LR, Hall CL, Richardson G, Jensen OE, Siggers JH, Foss AJE. Intracellular Flow in Optic Nerve Axons: A Mechanism for Cell Death in Glaucoma. Invest Ophth Vis Sci. 2009;50(8):3750-8. doi: 10.1167/iovs.08-2396. PubMed PMID: WOS:000268398000028.

8. Balaratnasingam C, Morgan WH, Johnstone V, Cringle SJ, Yu DY. Heterogeneous Distribution of Axonal Cytoskeleton Proteins in the Human Optic Nerve. Invest Ophth Vis Sci. 2009;50(6):2824-38. doi: 10.1167/iovs.08-3206. PubMed PMID: WOS:000266403800041.

9. Jonas JB, Holbach L. Central corneal thickness and thickness of the lamina cribrosa in human eyes. Invest Ophth Vis Sci. 2005;46(4):1275-9. doi: 10.1167/iovs.04-0851. PubMed PMID: WOS:000227908900024.

10. Chen H, Ding GS, Zhao YC, Yu RG, Zhou JX. Ultrasound measurement of optic nerve diameter and optic nerve sheath diameter in healthy Chinese adults. Bmc Neurol. 2015;15. doi: ARTN 106

10.1186/s12883-015-0361-x. PubMed PMID: WOS:000357374500001.

11. Rosen AM, Denharn DB, Fernandez V, Boria D, Ho A, Matins F, et al. In vitro dimensions and curvatures of human lenses. Vision Res. 2006;46(6-7):1002-9. doi: 10.1016/j.visres.2005.10.019. PubMed PMID: WOS:000236073400025.
