# Supplementary material for: Estimating three-dimensional outflow and pressure gradients within the human eye
Source: PLoS One. 2019 Apr 9;14(4):e0214961. doi: 10.1371/journal.pone.0214961 (PMC6456205; doi:10.1371/journal.pone.0214961)
Supplement: S2 Table — (DOCX) [file pone.0214961.s002.docx]

**S2 Table. Hydraulic conductivities of various tissues.**

| Hydraulic conductivity of Neuronal Tissues and Vitreous Humor | | | | |
| --- | --- | --- | --- | --- |
| Tissue | Species | Test type and comments | Estimated hydraulic conductivity (m^2^/Pa-s) | Reference |
| Experimentally measured data | | | | |
| retina | rabbit | laboratory—fresh and previously frozen tissue; thickness assumed to be 100 microns | at 37 degrees C 9.4×10^-12^± s.d. equals 2.3×10^-12^, giving a range of about 5.0×10^-12^ to 1.4×10^-11^ | [1] |
| retina | human | laboratory—fixed tissue mounted on cellulose membrane | Assuming retinal thickness 200 microns, then 5.0×10^-14^ ± s.d. = 2.0×10^-14^ | [2] |
| brain | rat | in-vivo—capsule around basal ganglia—white matter | 6.5×10^-11^ | [3] |
| brain | rat | in-vivo—caudate-putamen—gray matter | 2.1×10^-12^ | [3] |
| brain | rat | in vivo, gray matter | 2.1×10^-12^ | [4] |
| brain | cat | white matter | 1.6×10^-11^ | [5] based on experimental data gather by [6] |
| vitreous humor | bovine | laboratory | 8.4×10^-11^ ± s.d. = 4.5×10^-11^ | [7] |
| vitreous humor | Bovine/rabbit | laboratory | 3.0/6.0×10^-12^ | [8, 9] |
| Previous modeling parameter estimates | | | | |
| brain | NA | gray matter | 5×10^-12^ | [10] |
| brain | NA | white matter | 7.5×10^-12^ | [10] |
| brain | human | white matter | 1.4×10^-11^ | [11] |

**References**

1. Fatt I, Shantinath K. Flow Conductivity of Retina and Its Role in Retinal Adhesion. Exp Eye Res. 1971;12(2):218-+. doi: Doi 10.1016/0014-4835(71)90094-7. PubMed PMID: WOS:A1971K637300011.

2. Antcliff RJ, Hussain AA, Marshall J. Hydraulic conductivity of fixed retinal tissue after sequential excimer laser ablation - Barriers limiting fluid distribution and implications for cystoid macular edema. Arch Ophthalmol-Chic. 2001;119(4):539-44. PubMed PMID: WOS:000168130700007.

3. Nobrega TL. AN INFUSION-PRESSURE SYSTEM TO DETERMINE HYDRAULIC CONDUCTIVITY OF SOFT BIOLOGICAL TISSUES AND MONITOR CLINICAL INFUSIONS [Master of Engineering]: University of Florida; 2010.

4. Neeves KB, Lo CT, Foley CP, Saltzman WM, Olbricht WL. Fabrication and characterization of microfluidic probes for convection enhanced drug delivery. J Control Release. 2006;111(3):252-62. doi: 10.1016/j.jconrel.2005.11.018. PubMed PMID: WOS:000237153900002.

5. Kaczmarek M, Subramaniam RP, Neff SR. The hydromechanics of hydrocephalus: Steady-state solutions for cylindrical geometry. B Math Biol. 1997;59(2):295-323. PubMed PMID: WOS:A1997WL28000005.

6. Reulen HJ, Graham R, Spatz M, Klatzo I. Role of Pressure-Gradients and Bulk Flow in Dynamics of Vasogenic Brain Edema. J Neurosurg. 1977;46(1):24-35. doi: DOI 10.3171/jns.1977.46.1.0024. PubMed PMID: WOS:A1977CR17600003.

7. Xu J, Heys JJ, Barocas VH, Randolph TW. Permeability and diffusion in vitreous humor: Implications for drug delivery. Pharmaceut Res. 2000;17(6):664-9. doi: Doi 10.1023/A:1007517912927. PubMed PMID: WOS:000088665300005.

8. Fatt I. Flow and diffusion in the vitreous body of the eye. Bull Math Biol. 1975;37(1):85-90. PubMed PMID: 1120204.

9. Fatt I, Weissman BA. Physiology of the Eye: An Introduction to the Vegetative Functions. 2nd ed: Butterworth-Heineman; 1992.

10. Basser PJ. Interstitial Pressure, Volume, and Flow during Infusion into Brain-Tissue. Microvascular Research. 1992;44(2):143-65. doi: Doi 10.1016/0026-2862(92)90077-3. PubMed PMID: WOS:A1992JT39600002.

11. Vardakis JC, Tully BJ, Ventikos Y. Exploring the Efficacy of Endoscopic Ventriculostomy for Hydrocephalus Treatment via a Multicompartmental Poroelastic Model of CSF Transport: A Computational Perspective. Plos One. 2013;8(12). doi: ARTN e84577

10.1371/journal.pone.0084577. PubMed PMID: WOS:000329323900111.
