# Supplementary material for: Estimating three-dimensional outflow and pressure gradients within the human eye
Source: PLoS One. 2019 Apr 9;14(4):e0214961. doi: 10.1371/journal.pone.0214961 (PMC6456205; doi:10.1371/journal.pone.0214961)
Supplement: S3 Table — (DOCX) [file pone.0214961.s003.docx]

**S3 Table. Change in IOP from ODE model as components of the eye are sequentially added to the 3D PDE flow model.**

| **Change in IOP for constant aqueous production rate (initially 6.318 microlitres/min)** | | | | |
| --- | --- | --- | --- | --- |
|  | **Sequential updated parameter change** | **Best estimate (IOP in mmHg).** | **Minimum (IOP in mmHg)** | **Maximum(IOP in mmHg)** |
| Base Case 1: $\alpha$ equals 0.075; $C_{T}^{SL}$ equals 1.0, $p_{T}$ equals 3 mmHg and $p_{back}$ equals zero mmHg;  ODE model | NA | 15 | 15 | 15 |
| + retina  (base case 1  k_ret_=1×10^-5^) | k_ret_ = 5.0×10^-14^ | 15.56 | NA | NA |
|  | k_ret_= 5.0×10^-12^ | NA | 15.006 | NA |
|  | k_ret_= 1.0×10^-14^; k_ret_= 5.0×10^-14^;k_ret_=1.0×10^-13^; k_ret_= 1.0×10^-12^ | NA | NA | 17.70;15.56;  15.28;15.03 |
| + vitreous  base case  k_vit_=1×10^-5^) | k_vit_ = 8.4×10^-11^ | 15.61 | NA | NA |
|  | k_vit_ = 5×10^-10^ | NA | 15.02 |  |
|  | k_vit_ = 1.0×10^-11^ | NA | NA | 18.11 |
| + flow through optic nerve head (base case 1  k_ONLC_=1.0×10^-18^) | k_ONLC_ = 1.0×10^-14^ | 15.59 |  |  |
|  | k_ONLC_ = 1.0×10^-15^ | NA | NA | 18.11 |
|  | k_ONLC_ = 1.0 ×10^-13^ | NA | 14.94 | NA |
| Final IOP | NA | 15.59 | 14.94 | 18.11 |
| Total change IOP | NA | 0.59 | -0.06 | 3.11 |
| % change IOP | NA | 3.8% | -0.4% | 17% |
| Pressure drop front to back eye | NA | 0.14 | 0.11 | 1.0 |
| % pressure drop front to back of eye | NA | 1.0% | 0.74% | 5.5% |
| Change in aqueous production rate (initially 6.32 microlitres/min) for constant IOP 15 mmHg | | | | |
| Change in aqueous production rate | NA | -0.19 | 0.03 | -0.90 |
| % change in aqueous production rate | NA | -3.0% | 0.5% | -14.3% |

Note: details of all references are given at the end of the paper.
